# Two-Step Synthesis, Structure, and Optical Features of a Double Hetero[7]helicene

**DOI:** 10.3390/molecules27249068

**Published:** 2022-12-19

**Authors:** Mohamed S. H. Salem, Ahmed Sabri, Md. Imrul Khalid, Hiroaki Sasai, Shinobu Takizawa

**Affiliations:** 1 SANKEN, Osaka University, Ibaraki-shi, Osaka 567-0047, Japan; 2 Pharmaceutical Organic Chemistry Department, Faculty of Pharmacy, Suez Canal University, Ismailia 41522, Egypt; 3 Graduate School of Pharmaceutical Sciences, Osaka University, Suita-shi, Osaka 565-0871, Japan

**Keywords:** polycyclic aromatic hydrocarbon, double hetero[7]helicene, short-step synthesis, electrochemical cross-coupling, nucleus-independent chemical shift

## Abstract

A novel double aza-oxa[7]helicene was synthesized from the commercially available *N^1^*,*N^4^*-di(naphthalen-2-yl)benzene-1,4-diamine and *p*-benzoquinone in two steps. Combining the acid-mediated annulation with the electrochemical sequential reaction (oxidative coupling and dehydrative cyclization) afforded this double hetero[7]helicene. Moreover, the structural and optical features of this molecule have been studied using X-ray crystallographic analysis, and the absorption and emission behaviors were rationalized based on DFT calculations.

## 1. Introduction

**Figure 1:**
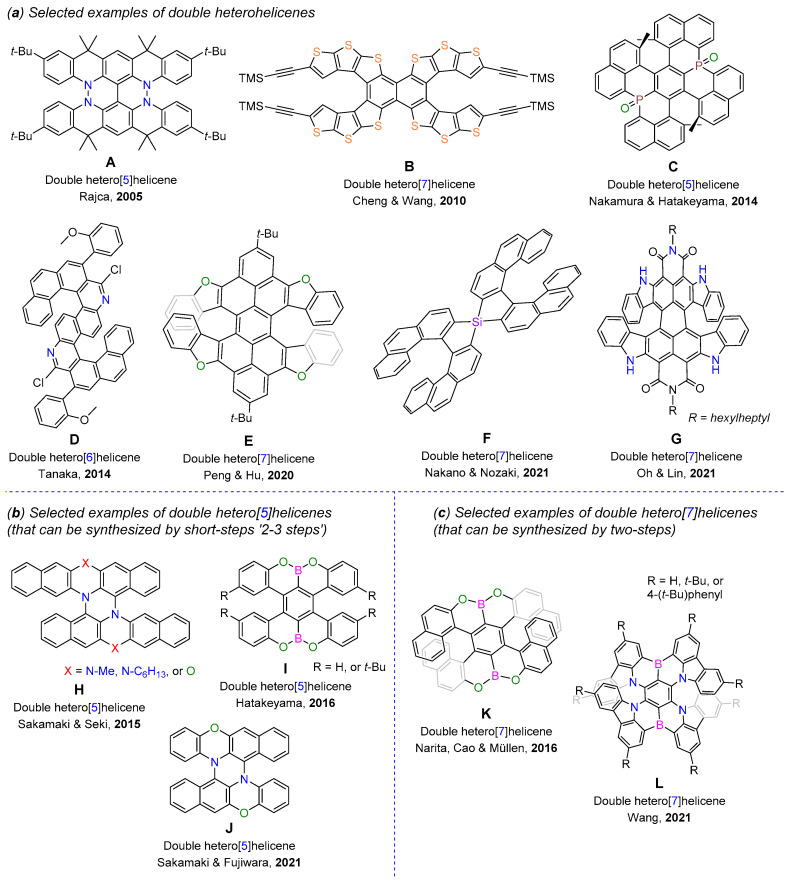
The selected examples of double heterohelicenes in short-step synthesis: (**a**) Double hetero[5–7]helicenes (more than four-step synthesis); (**b**) Double hetero[5]helicenes (two- or three-step synthesis); (**c**) Double hetero[7]helicenes (two-step synthesis).

Helicenes are polycyclic aromatic hydrocarbons (PAHs) in which aromatic rings are annulated in a helical architecture, giving them unique electronic, photophysical and chiroptical properties [1,2,3,4]. Over the past couple of decades, the great advances achieved in this chemistry [2,3,4,5,6] promoted a broad spectrum of material-based applications [7,8,9,10], transistors [11,12], and semiconductors [13]. Incorporation of one or more heteroatoms in the helicene scaffolds modulate their physical and optical features, and alter the electronic properties in order to expand their applications [14,15]. With these extra features, the trend in helicene chemistry has begun to move towards heterohelicenes after the domination of carbohelicenes [16,17,18,19,20,21]. Another approach to promote characteristics of helicenes is to induce multihelicity which means combining two or more helical scaffolds in a single molecule [22,23]. Multiple helicenes show a lot of favorable properties due to their amplified non-planarity, diverse conformations, and maximized intermolecular interactions [24,25]. Various smart core scaffolds were used to induce this multihelicity such as perylene diimide (PDI) that afforded valuable twisted structures for different material-based applications [26,27,28,29]. Hence, a lot of efforts were dedicated for designing and synthesizing multiple heterohelicenes [30], in particular, double heterohelicenes [14]. After the first report of double helicene reported by Rajca, many examples of these double heterohelicenes were conducted and exhibited clear superiority over their single counterparts, especially in terms of optical properties (Figure 1a) [31,32,33,34,35,36,37,38,39,40]. However, during that frantic pursuit to promote the properties of helicenes, another problem, in particular, synthetic difficulty emerged. With the increase in structural complexity, the synthesis of multiple heterohelicenes becomes more challenging and requires many steps. Although few reports succeeded to introduce effective short-step synthetic protocols for double heterohelicene, most of these successes were concentrated in the double hetero[5]helicene derivatives (Figure 1b) [41,42,43]. In 2016, Narita, Cao, and Müllen introduced an efficient two-step synthesis of a highly strained OBO-fused double hetero[7]helicene **K** via the nucleophilic aromatic substitution reaction of hexabromobenzene, followed by a sequential step of demethylation and C-H aryl borylation (Figure 1c) [44]. Earlier in the same year, Hatakeyama showed the potential of this synthetic approach to afford their boron-fused double hetero[5]helicene **I** (Figure 1b) [42]. In 2021, Wang and coworkers developed the first examples of B,*N*-embedded double hetero[7]helicenes **L** that showed excellent chiroptical features in the visible range [45]. With only two steps, they succeeded to prepare this double hetero[7]helicene **L** via the nucleophilic aromatic substitution of dibromotetrafluorobenzene with carbazole, followed by a tandem process of substitution with BBr_3_ and C-H aryl borylation [45].

Notably, these examples (Figure 1c) represent a quantum leap in the short-step synthesis of double hetero[7]helicenes via the tandem process of nucleophilic substitution with BBr_3_ followed by C-H aryl borylation [44,45]. As part of our effort to explore the electrochemical domino syntheses, we were interested in designing effective sequential reactions to access double helicene motifs [46,47]. Herein, a facile preparation of a double aza-oxa[7]helicene with a phenylene linker has been established through acid-mediated annulation with the electrochemical sequential reaction (oxidative coupling and dehydrative cyclization). We also studied the structural and optical features via x-ray crystallographic analysis, spectrophotometric analysis, and DFT calculations.

## 2. Results and Discussion

### 2.1. Synthesis of Double Aza-oxa[7]helicene **3**

**Scheme 1:**
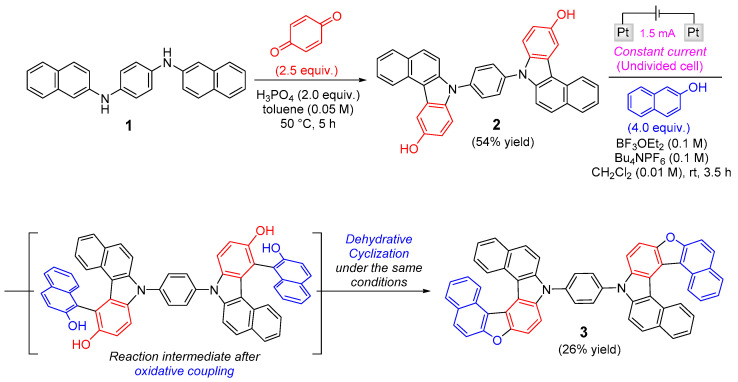
The synthesis of a double aza-oxa[7]helicenes **3**.

Recently, Zhang reported a facile acid-mediated synthesis of carbazole in which aniline derivatives react with *p*-benzoquinone to produce 3-hydroxycarbazoles [48]. Combining this approach with our unprecedented electrochemically enabled synthesis of hetero[7]helicenes and dehydro-hetero[7]helicenes [46,47], herein, we achieved the two-step synthesis of double aza-oxa[7]helicenes as depicted in Scheme 1. The acid-mediated annulation of the commercially available substrates; *N^1^*,*N^4^*-di(naphthalen-2-yl)benzene-1,4-diamine **1** and *p*-benzoquinone afforded the corresponding bis-3-hydroxy-benzo[c]carbazole **2** in 54% yield via a tandem process of double Michael addition and subsequent double ring closure. Next, a DCM solution of **2**, β-naphthol, and tetrabutylammonium hexafluorophosphate(V) as an electrolyte, was utilized to a constant current of 1.5 mA in an undivided electrolysis cell with platinum electrodes for 3.5 h at room temperature, affording double aza-oxa[7]helicene **3** in 26% yield. The electrochemical sequential synthesis of **3** proceeds through the oxidative hetero-coupling of arenols to produce a diol intermediate that can readily undergo a subsequent dehydrative cyclization to **3**. All compounds showed good chemical and thermal stabilities and no decomposition was observed upon purification on silica column chromatography and heating at 100 °C in air.

### 2.2. Structure and Packing Mode of **3**

**Figure 2:**
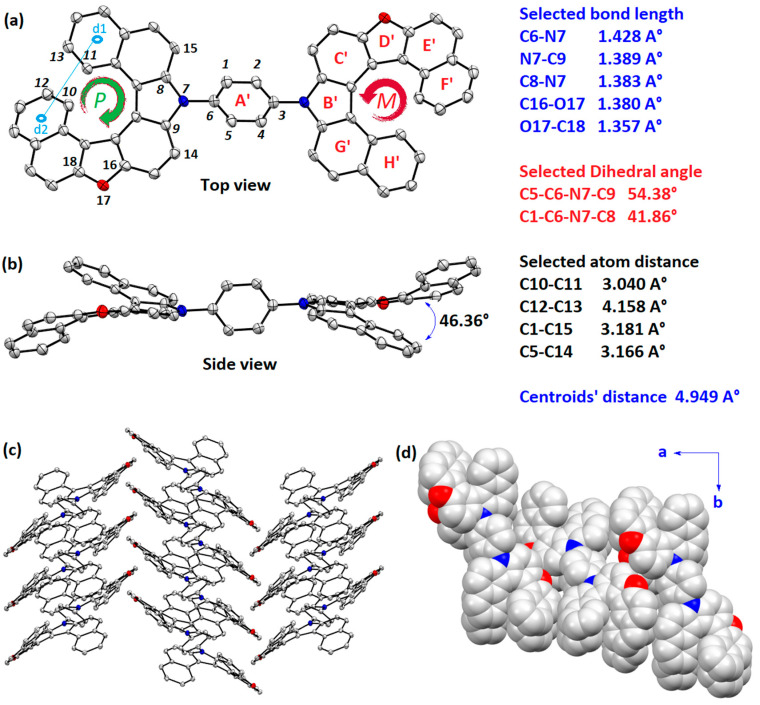
Single crystal structure of the double helicene **3**: (**a**,**b**) ORTEP drawing structure of (*P*,*M*)-**3** obtained by X-ray crystal analysis with ellipsoids at 50% probability (H atoms were omitted for clarity); (**c**) crystal packing of (*P*,*M*)-**3** with ellipsoids at 30% probability; (**d**) packing structure of (*P*,*M*)-**3** is viewed along the c-axis to show the herringbone arrangement.

The double aza-oxa[7]helicene structure of **3** was definitely confirmed by X-ray crystallography using a single crystal, grown from its racemic solution. We used the liquid/liquid diffusion technique between ethyl acetate and *n*-hexane to prepare this crystal slowly over three days in a dark environment at −20 °C. As expected, the two helicene moieties are connected via a phenylene linker (Figure 2a,b). The dihedral angles between the phenylene linker’s plane and the pyrrole (ring B’) are −41.86° for (C_1_-C_6_-N_7_-C_8_), and 54.38° for (C_5_-C_6_-N_7_-C_9_). Although the experimental values of (C_5_-C_6_-N_7_-C_9_) dihedral angle (54.38°) is comparable to that of the optimized structure using DFT calculations at MN15/6-311G(d,p) level of theory (54.72°), (C_1_-C_6_-N_7_-C_8_) dihedral angle was smaller than optimized structures at various levels (Table 1). This can be attributed to the intermolecular interactions between the double helicene molecules **3** in the packed structure. Only meso isomer (*P*,*M*)-**3** was observed in the crystal packing with achiral molecules packed along the b-axis (Figure 2c,d). The packing of **3** shows a herringbone pattern with π-π distance of 4.458 A°. This characteristic arrangement is optimum for many material-based applications, especially semiconductors [49,50,51]. In addition, it maximizes the optical and electronic properties of the obtained double helicene upon self-assembly [49,52,53,54]. Most of these larger or multiple helicenes showed significant variations during DFT calculations owing to the long-range conjugation and the effects of charge transfer [55,56]. Among the functions we screened, Minnesota 15 (MN15) function was found to be the most suitable parameters for our molecules (Table 1) [57].

**Figure 3:**
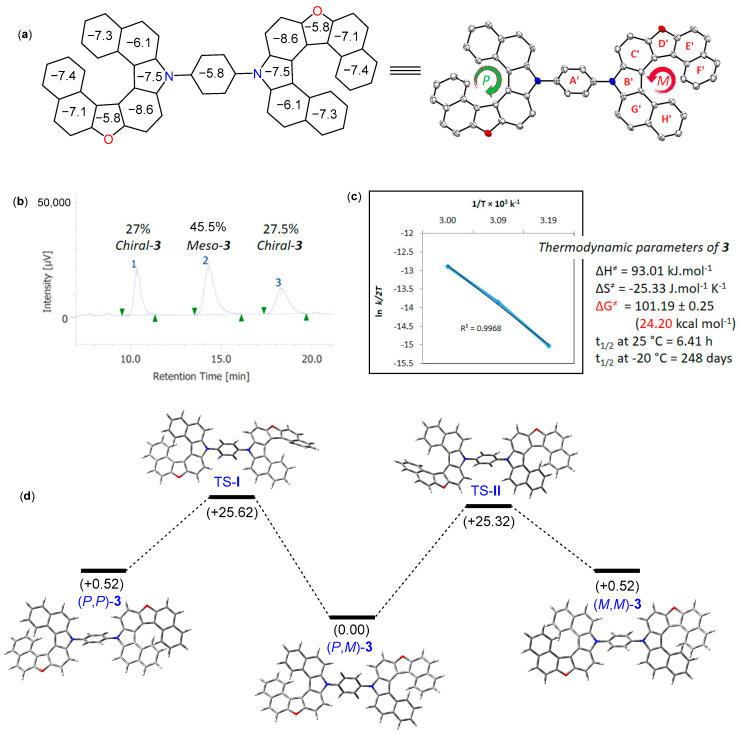
(**a**) NICS (0) values of (*P*,*M*)-**3** calculated at the MN15/6-311G+(2d,p) level; (**b**) HPLC chromatogram determined by (Daicel Chiralpak IA, *n*-hexane/*i*-PrOH = 20/1, flow rate 1.0 mL/min, T = 25 ºC, 240 nm): t_1_= 10.36 min, t_2_ = 14.30 min, and t_3_ = 18.32 min; (**c**) Eyring plot of compound **3** epimerization and thermodynamic parameters; (**d**) Epimerization process from (*P*,*M*)-**3** isomer to (*M*,*M*)-**3** and (*P*,*P*)-**3** isomers. The relative Gibbs free energies are calculated in (kcal mol^−1^) at the MN15/6-311G(d,p) level.

Nucleus-independent chemical shift (NICS) calculations revealed the low aromaticity of the central phenylene linker with a NICS (0) value of −5.8 ppm (Figure 3a), much lower than that of benzene −7.6 ppm calculated at the same level of theory. The largest NICS (0) values (between −7.3 ppm and −8.6 ppm) were found on the benzene of 6*H*-furo[3,2-*e*]indole (ring C′), pyrrole (ring B′) and naphthalene (rings F’ and H’). While the lowest NICS (0) values (around −5.8 ppm) were found on the phenylene linker (ring A’) and furan rings (D’) which is consistent with the aromatic character of this ring. Generally, symmetric double hetero[*n*]helicenes (*n* ≥ 4) have three isomers, those being two chiral enantiomers (*P*,*P*) and (*M*,*M*), and one meso diasteromer (*P*,*M*) [30]. All three isomers of **3** were afforded under our reaction conditions which was confirmed by HPLC separation using DAICEL CHIRALPAK IA column (eluent: *n*-hexane/*i*-PrOH = 20/1) (Figure 3b). The experimental ratio among the three isomers was found to be around (1:2:1) with the meso isomer (*P*,*M*)-**3** as the major formed product (confirmed by the absence of optical rotation). After HPLC chiral resolution, the epimerization rate of **3** was studied at three different temperatures (See SI). Eyring plot (Figure 3c) indicated a low chiral stability of **3** (epimerization barrier ~24.2 kcal mol^−1^) with an estimated half-life of the epimerization <6.5 h at 25 °C. These observations were matching with our DFT calculations that showed similar epimerization barriers 25.32 kcal mol^−1^ and 25.62 kcal mol^−1^ (Figure 3d).

### 2.3. Photophysical Properties

**Figure 4:**
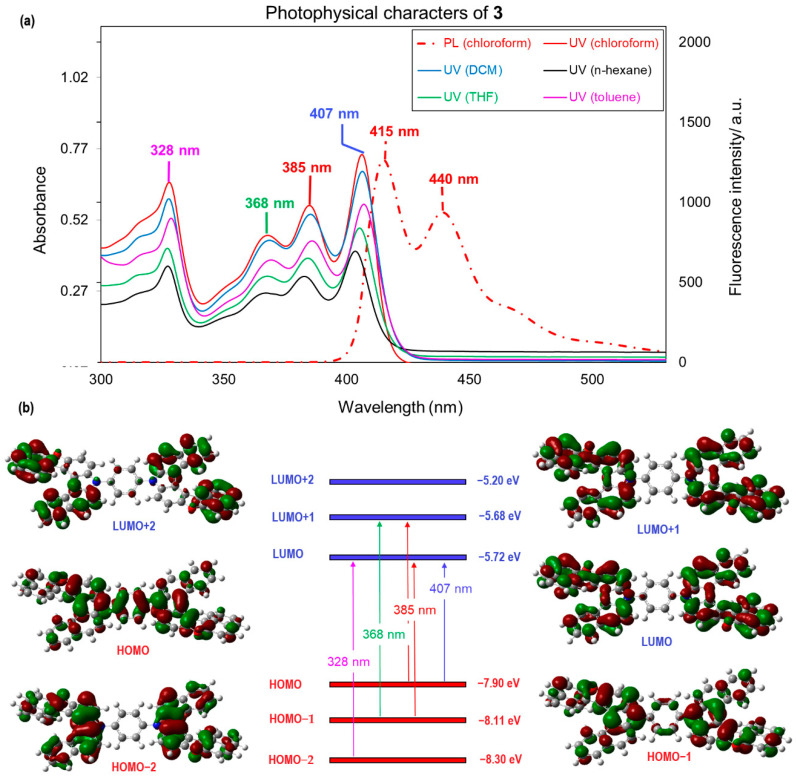
(**a**) UV/vis absorption and PL spectra of **3** in various solvents (20 µM); (**b**) Frontier Kohn-Sham molecular orbitals of **3** and TD-DFT calculated electronic transitions at MN15/6-311G (d,p) level of theory.

Our double aza-oxa[7]helicene **3** shows high luminescence upon photo-irradiation, which can be attributed to the rigid scaffold that hinders the thermal energy loss upon structural changes. The UV/vis absorption of **3** was recorded in different solvents to show its high solubility in most of the organic solvents which increases the potential for some applications that require good solubility such as solution-processed electronics [58,59,60]. In all measured solvents, compound **3** showed similar UV/vis absorption patterns (Figure 4a). The maximum absorbance exhibited in chloroform was shown at 407 nm (absorption coefficient: 7.5 × 10^4^ M^−1^ cm^−1^) corresponding to an optical energy gap of (2.18 eV). According to TD-DFT calculations at the MN15/6-311G(d,p) level of theory, this low-energy absorption can be accountable to the HOMO→LUMO transition. The absorption band at 385 nm possibly attributed to the equal contribution of both HOMO−1→LUMO and HOMO→LUMO+1 transitions. The band at 368 nm is estimated to be due to the HOMO−1→LUMO+1 transition, while the higher energy absorption band at 328 nm would be attributed to HOMO−2→LUMO corresponding to an optical energy gap of (2.58 eV). Molecular orbital calculations indicated that the HOMO is spread mainly over the phenylene linker (ring A′) and pyrroles (rings B’) and LUMO is spread over the whole scaffold rather than the phenylene linker (ring A′), accounting for the substantial stability. Photoluminescence PL spectrum of **3** was recorded in a pure chloroform solution exhibiting emission maxima shifted in a bathochromic way at 415 nm and 440 nm.

### 2.4. Energetic Characterization by Cyclic Voltammetry

**Figure 5:**
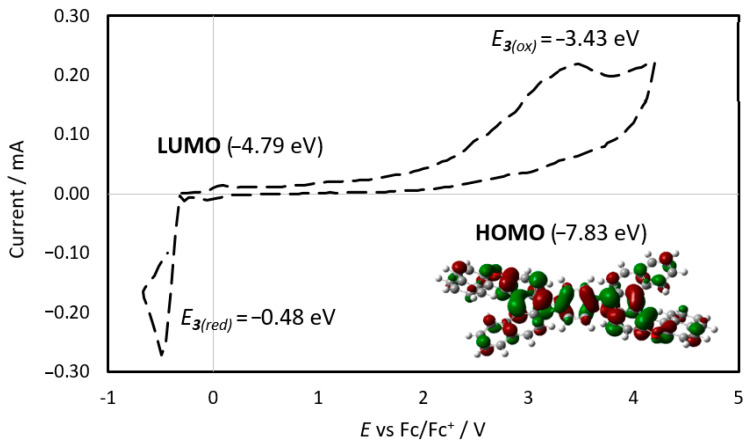
The cyclic voltammetry profile of **3** in (MeCN) with *n*-Bu_4_NPF_6_ (0.1 M) using ferrocene as internal reference.

Cyclic voltammetry (CV) measurements of our double aza-oxa[7]helicene **3** showed reversible redox peaks in both negative and positive regions indicating the chemical stability of its anion/cation pairs and how they can be reduced or oxidized readily to the neutral form (Figure 5). Using ferrocene and ferrocenium as internal references, the HOMO energy level of **3** was calculated using Bredas empirical equation to be around (–7.83 eV) which is comparable to the DFT-calculated HOMO energy (−7.90 eV) [61]. *E*_LUMO_ could be estimated after considering the gap between *E*_HOMO_ and *E*_LUMO_ (3.04 eV) from the λ_max_ or excitation energy (407 nm) to be around (−4.79 eV) showing little higher energy than the DFT-calculated LUMO (−5.72 eV).

## 3. Materials and Methods

### 3.1. General Experimental Details

^1^H-, and ^13^C-NMR were recorded via JNM ECA600 FT NMR (^1^H-NMR 600 MHz, ^13^C-NMR 151 MHz). ^1^H-NMR spectra are reported as follows: a chemical shift in ppm downfield of tetramethylsilane (TMS) and referenced to residual solvent peak (CDCl_3_) at 7.26 ppm, or ((CD_3_)_2_CO) at 2.05 ppm, multiplicities (s = singlet, d = doublet, dd = doublet of doublets, t = triplet, q = quartet, m = multiplet), and coupling constants (Hz). ^13^C-NMR spectra reported in ppm relative to the central line of triplet for CDCl_3_ at 77.16 ppm, or the central line of septet for ((CD_3_)_2_CO) at 29.84 ppm. APCI-MS spectra were obtained with JMS-T100LC (JEOL). FT-IR spectra were recorded on a JASCO FT-IR system (FT/IR4100). Photoluminescence (PL) spectra were recorded on JASCO FP-8550 Spectrofluorometer. UV spectra were recorded on a JASCO v-770 spectrophotometer. Column chromatography on SiO_2_ was performed with Kanto Silica Gel 60 (63–210 μm). Commercially available organic and inorganic compounds were used without further purification. The electro-oxidation was carried out using sing ElectraSyn^®^ 2.0 (designed by IKA) at a constant current of 1.5 mA, under air (1 atm.) [62].

### 3.2. Synthetic Procedures

#### 3.2.1. General Procedure for the Synthesis of Double 3-Hydroxy Benzo[c]carbazole **2**

To a solution of *N^1^*,*N^4^*-di(naphthalen-2-yl)benzene-1,4-diamine **1** (36 mg, 0.1 mmol) and *p*-benzoquinone (27 mg, 0.25 mmol, 2.5 equiv.) in dry toluene (1.5 mL), orthophosphoric acid (10.6 µL, 2.0 equiv.) dissolved in (0.5 mL) toluene was added dropwise. The reaction mixture was stirred at 50 °C for 5 h under N_2_ atmosphere until its completion. Next, the reaction was quenched via water, extracted with EtOAc and the combined organic extracts dried over Na_2_SO_4_, and evaporated *in vacuo*. The crude mixture was purified on silica column chromatography (eluent: *n*-hexane/DCM/ethyl acetate = 7/1/1) to give double 3-Hydroxy benzo[c]carbazole **2** as a white solid in 54% yield.

- 7,7’-(1,4-Phenylene)bis(*7H*-benzo[*c*]carbazol-10-ol) **2**

^1^H NMR (600 MHz, (CD_3_)_2_CO): *δ* 8.80 (d, *J* = 8.2 Hz, 2H), 8.31 (s, 2H), 8.17 (d, *J* = 2.1 Hz, 2H), 8.07 (d, *J* = 8.2 Hz, 2H), 7.92–7.96 (m, 6H), 7.75–7.78 (m, 4H), 7.58 (d, *J* = 8.9 Hz, 2H), 7.50 (dd, *J* = 8.3, 6.9 Hz, 2H), 7.14 (dd, *J* = 8.6, 2.4 Hz, 2H); ^13^C NMR (151 MHz, (CD_3_)_2_CO): *δ* 153.39, 139.70, 137.52, 135.41, 130.82, 130.43, 130.13, 129.80, 128.33, 127.95, 125.47, 123.91, 123.75, 115.93, 115.05, 112.67, 111.86, 107.78; DEPT-135 NMR (151 MHz, (CD_3_)_2_CO): *δ* 130.12, 129.80, 128.32, 127.95, 123.91, 123.74, 115.03, 112.67, 111.86, 107.76; HRMS (APCI): calcd for C_38_H_24_N_2_O_2_: m/z 541.1911 [M + H]^+^, found 541.1912.; IR (KBr): 3334, 3042, 2977, 2926, 1620, 1517, 1473, 1165, 831, 803 cm^−1^; mp: 198–199 °C.

#### 3.2.2. General Procedure for the Synthesis of Double Aza-oxa[7]helicene **3**

A 10 mL DCM solution of **2** (54 mg, 0.1 mmol), β-naphthol (57.7 mg, 0.4 mmol), tetrabutylammonium hexafluorophosphate(V) (387.4 mg, 1.0 mmol), and BF_3_OEt_2_ (0.2 M) was transferred into the undivided electrolysis cell of ElectraSyn^®^ 2.0. This cell is equipped with two Pt electrodes connected to a DC power supply. At room temperature, a constant current of 1.5 mA was applied for 3.5 h. After the completion of reaction, the electrolysis was stopped and crude mixture was purified by column chromatography (SiO_2_, EtOAc/*n*-hexane) to afford the double aza-oxa[7]helicene **3** as a yellow solid in 26% yield.

- 1,4-Bis(*10H*-benzo[*c*]naphtho[1’,2’:4,5]furo[3,2-*g*]carbazol-10-yl)benzene **3**

^1^H NMR (600 MHz, CDCl_3_): *δ* 8.38 (d, *J* = 8.2 Hz, 2H), 8.31 (d, *J* = 8.2 Hz, 2H), 8.00–8.04 (m, 12H), 7.92 (d, *J* = 8.9 Hz, 2H), 7.85 (d, *J* = 8.9 Hz, 4H), 7.77 (d, *J* = 8.9 Hz, 2H), 7.34–7.39 (m, 4H), 6.95–7.00 (m, 4H); ^13^C NMR (151 MHz, CDCl_3_): *δ* 154.75, 153.31, 138.79, 137.93, 137.32, 130.86, 129.79, 129.73, 129.50, 129.15, 128.59, 128.10, 128.05, 128.03, 125.13, 124.67, 124.37, 123.62, 120.07, 117.98, 117.77, 116.64, 112.65, 111.58, 109.45, 109.06 (Two carbons overlapped); DEPT-135 NMR (151 MHz, CDCl_3_): *δ* 129.79, 129.15, 128.59, 128.09, 128.05, 128.03, 125.13, 124.67, 124.37, 123.61, 112.66, 111.58, 109.44, 109.06 (One carbon overlapped); HRMS (APCI): calcd for C_58_H_32_N_2_O_2_: *m/z* 789.2537 [M + H]^+^, found 789.2542; IR (KBr): 3043, 2926, 2856, 1714, 1594, 1500, 1417, 1355, 1209, 805 cm^−1^; mp: 291–292 °C.

### 3.3. DFT Calculations

All DFT calculations were performed using the Gaussian 16 package of programs [63]. The geometries of the model compounds were optimized using three DFT functions: B3LYP, wB97XD, and MN15 at 6-311G(d,p) basis set. All stationary points were identified as stable minima by frequency calculations. Geometry optimization was achieved using the normal criteria defined in Gaussian 16. TD-DFT calculations were performed using two different levels of theory B3LYP/6-311G(d,p) and MN15/6-311G(d,p) in both chloroform and gas-phase. All structures were optimized without any symmetry assumptions. For further computational details, see Supplementary Materials.

## 4. Conclusions

In summary, we introduced a two-step protocol to synthesize double aza-oxa[7]helicene **3** using an electrochemical approach. This novel double hetero[7]helicene shows interesting structural features that were reflected in its excellent optical properties. We have studied the photophysical characteristics of this compound and correlated its absorption and fluorescence behavior based on DFT calculations. Further development for this two-step protocol towards the preparation of other multiple helicenes and PHAs and study their photophysical and chiroptical features are currently under investigation.

## Figures and Tables

**Table 1:** The selected experimental and calculated structural parameters of double aza-oxa[7]helicene **3**.

Parameters	Experimental	B3LYP ^1^	wB97XD ^1^	MN15 ^1^
Centroids’ distance (rings F’-H’)	4.949 A°	4.885 A°	4.721 A°	4.759 A°
d_1_-N_7_-d_2_ Centroid angle	46.36°	45.43°	44.24°	45.51°
C_5_-C_6_-N_7_-C_9_ Dihedral angle	54.38°	60.25°	59.03°	54.72°
C_1_-C_6_-N_7_-C_8_ Dihedral angle	41.86°	57.08°	55.34°	51.42°
C_1_-C_15_ Distance	3.181 A°	3.316 A°	3.281 A°	3.241 A°
C_5_-C_14_ Distance	3.166 A°	3.356 A°	3.316 A°	3.266 A°

^1^ All calculations are carried out using 6-311G(d,P) basis set at three different functions (B3LYP, wB97XD, and MN15).

## Data Availability

CIF of the crystal of **3** is available as Supplementary. The X-ray crystallographic coordinate for the structure reported in this study has been deposited at the Cambridge Crystallographic Data Centre (CCDC) under deposition numbers CCDC-2156335 (**3**). These data can be obtained free of charge from The Cambridge Crystallographic Data Centre via www.ccdc.cam.ac.uk/data_request/cif.

## Supplementary Materials

The following supporting information can be downloaded at: https://www.mdpi.com/article/10.3390/molecules27249068/s1, Table S1: Optimization of the acid-mediated annulation step; Table S2: Optimization of the electrochemical sequential reaction; Table S3: Selected experimental and calculated structural parameters of double aza-oxa[7]helicene **3**; Tables S4–S6: Summary of the TD-DFT calculation results of **3**; Scheme S1: A plausible mechanism for the electrochemical domino reaction; Figure S1: Crystal measurements; Figure S2: Measurements of the optimized structures; Figure S3: Selected molecular orbitals of **3**; Figure S4: Simulated UV-vis absorption and CD spectra of (*P*,*M*)-**3**; Figure S5: Further NICS(0) calculations [57,63,64,65,66,67,68].

## Sample Availability

Samples of compound **3** are available from the corresponding author upon reasonable request.
