# Supplementary material for: Two-Step Synthesis, Structure, and Optical Features of a Double Hetero[7]helicene
Source: Molecules. 2022 Dec 19;27(24):9068. doi: 10.3390/molecules27249068 (PMC9785389; doi:10.3390/molecules27249068)
Supplement: Supplementary file 1 [file molecules-27-09068-s001.zip › molecules-2097390-supplementary.pdf]

Supporting Information

Two-Step Synthesis, Structure, and Optical Features of a Double Hetero[7]helicene

Mohamed S. H. Salem [1,2], Ahmed Sabri [1], Md. Imrul Khalid [1], Hiroaki Sasai [1,3] and Shinobu Takizawa [1,*]

[1] *SANKEN, Osaka University, Ibaraki-shi, Osaka 567-0047, Japan*

[2] *Pharmaceutical Organic Chemistry Department, Faculty of Pharmacy, Suez Canal University, Ismailia 41522, Egypt*

[3] *Graduate School of Pharmaceutical Sciences, Osaka University, Suita-shi, Osaka 565-0871, Japan*

E-mail: taki@sanken.osaka-u.ac.jp

Tel: +81-6-6879-8467; Fax: +81-6-6879-8469

Table of Contents

1.	X-ray crystallographic analysis	S2
2.	Optimization of the two-step synthetic protocol	S4
3.	A plausible mechanism for the electrochemical domino reaction	S6
4.	HPLC chromatogram	S7
5.	NMR Spectra	S7
6.	Epimerization barrier study of **3**	S13
7.	DFT calculations	S14
8.	References	S36

1. X-ray crystallographic analysis

(*P*,*M*)-**3** (CCDC 2156335) with ellipsoids at 50% probability, (H atoms were omitted for clarity).

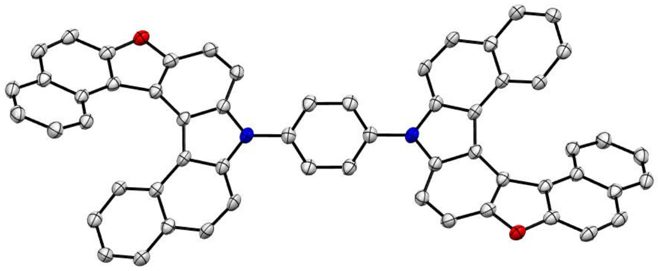

Empirical formula	$C_{58}H_{32}N_2O_2$
Formula weight	788.85
Temperature/K	293(2)
Crystal system	monoclinic
Space group	I2/a
a/Å	22.2530(10)
b/Å	4.4560(2)
c/Å	37.5710(18)
α/°	90
β/°	91.972(4)
γ/°	90
Volume/Å^3	3723.3(3)
Z	3
ρ_{calc}g/cm^3	1.055
μ/mm^{-1}	0.499
F(000)	1230.0
Crystal size/mm^3	0.09 × 0.08 × 0.05
Radiation	Cu Kα (λ = 1.54184)
2Θ range for data collection/°	4.548 to 151.27
Index ranges	$-27 \leq h \leq 26$, $-5 \leq k \leq 5$, $-45 \leq l \leq 47$
Reflections collected	9444
Independent reflections	3666 [R_{int} = 0.0383, R_{sigma} = 0.0490]
Data/restraints/parameters	3666/0/281
Goodness-of-fit on F^2	1.065
Final R indexes [I>=2σ (I)]	R_1 = 0.0899, wR_2 = 0.2313
Final R indexes [all data]	R_1 = 0.1155, wR_2 = 0.2495
Largest diff. peak/hole / e Å^{-3}	0.51/-0.36

Bond precision:	C-C = 0.0059 A		Wavelength=1.54184
Cell:	a=22.253(1)	b=4.4560(2)	c=37.5710(18)
	alpha=90	beta=91.972(4)	gamma=90
Temperature:	293 K		

	Calculated	Reported
Volume	3723.3(3)	3723.3(3)
Space group	I 2/a	I 1 2/a 1
Hall group	-I 2ya	-I 2ya
Moiety formula	C58 H32 N2 O2	1.333(C58 H32 N2 O2)
Sum formula	C58 H32 N2 O2	C77.33 H42.67 N2.67 O2.67
Mr	788.86	1051.81
Dx,g cm-3	1.407	1.407
Z	4	3
Mu (mm-1)	0.665	0.665
F000	1640.0	1640.0
F000'	1644.60	
h,k,lmax	27,5,47	27,5,47
Nref	3875	3666
Tmin,Tmax	0.942,0.967	0.772,1.000
Tmin'	0.942	

Correction method= # Reported T Limits: Tmin=0.772 Tmax=1.000
AbsCorr = MULTI-SCAN

Data completeness= 0.946 Theta(max)= 75.635

R(reflections)= 0.0899(2674) wR2(reflections)= 0.2495(3666)

S = 1.065 Npar= 281

The following ALERTS were generated. Each ALERT has the format
test-name_ALERT_alert-type_alert-level.
Click on the hyperlinks for more details of the test.

Alert level C

```
PLAT340_ALERT_3_C Low Bond Precision on  C-C Bonds ...............    0.00594 Ang.
PLAT906_ALERT_3_C Large K Value in the Analysis of Variance ......      4.433 Check
PLAT911_ALERT_3_C Missing FCF Refl Between Thmin & STh/L=    0.600         62 Report
```

Alert level G

```
FORMU01_ALERT_1_G  There is a discrepancy between the atom counts in the
            _chemical_formula_sum and _chemical_formula_moiety. This is
            usually due to the moiety formula being in the wrong format.
            Atom count from _chemical_formula_sum:    C77.33 H42.67 N2.67 O2.67
            Atom count from _chemical_formula_moiety:C77.31399 H42.65599 N2.666 O2
PLAT045_ALERT_1_G Calculated and Reported Z Differ by a Factor ...      1.333 Check
PLAT072_ALERT_2_G SHELXL First  Parameter in WGHT  Unusually Large       0.12 Report
PLAT083_ALERT_2_G SHELXL Second Parameter in WGHT  Unusually Large      15.82 Why ?
PLAT199_ALERT_1_G Reported _cell_measurement_temperature ..... (K)        293 Check
PLAT200_ALERT_1_G Reported   _diffrn_ambient_temperature ..... (K)        293 Check
PLAT398_ALERT_2_G Deviating  C-O-C   Angle From 120 for O001     .      105.5 Degree
PLAT720_ALERT_4_G Number of Unusual/Non-Standard Labels ..........         47 Note
PLAT870_ALERT_4_G ALERTS Related to Twinning Effects Suppressed ..          ! Info
PLAT912_ALERT_4_G Missing # of FCF Reflections Above STh/L=  0.600        147 Note
PLAT933_ALERT_2_G Number of HKL-OMIT Records in Embedded .res File         36 Note
PLAT941_ALERT_3_G Average HKL Measurement Multiplicity ...........        2.4 Low
PLAT955_ALERT_1_G Reported (CIF) and Actual (FCF) Lmax Differ by .          1 Units
```

```
 0 ALERT level A = Most likely a serious problem - resolve or explain
 0 ALERT level B = A potentially serious problem, consider carefully
 3 ALERT level C = Check. Ensure it is not caused by an omission or oversight
13 ALERT level G = General information/check it is not something unexpected
```

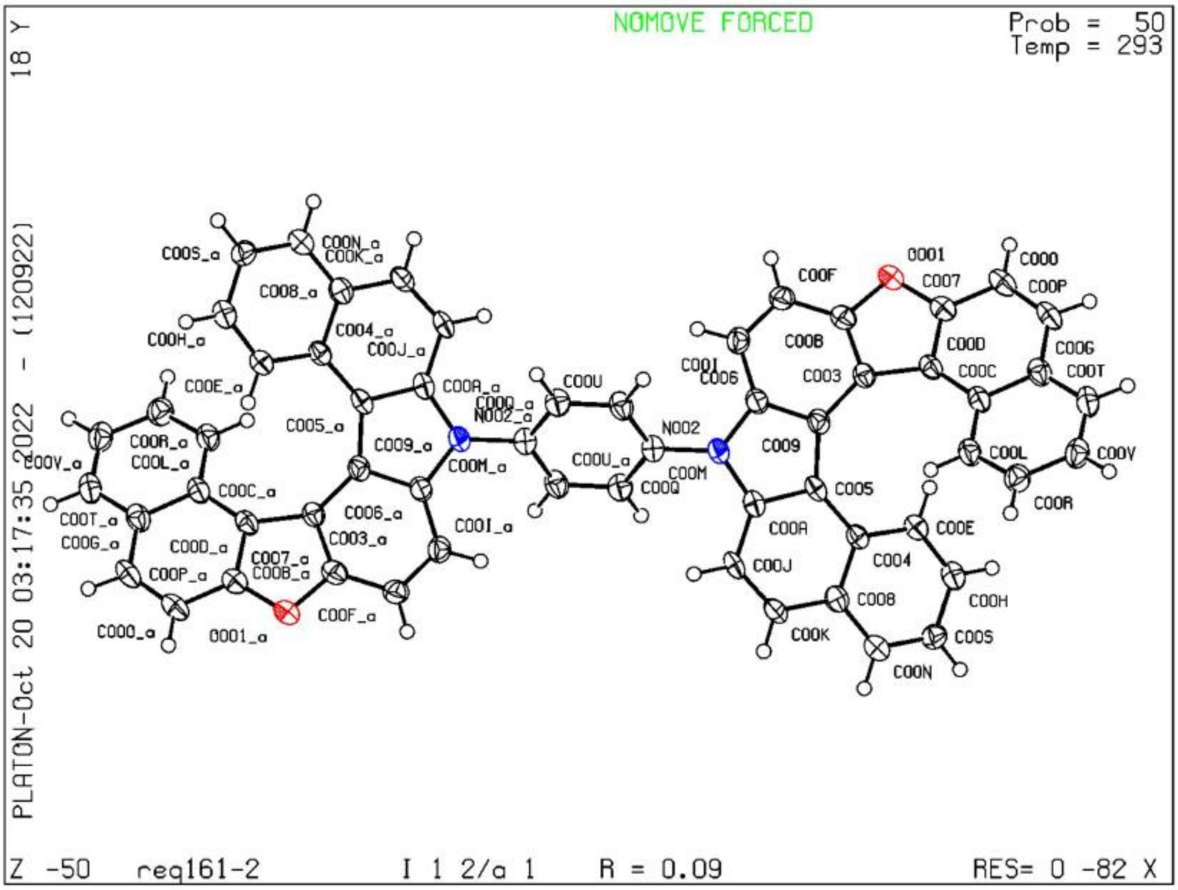

2. Rough optimization of the two-step synthetic protocol

Table S1. Optimization of the acid-mediated annulation step

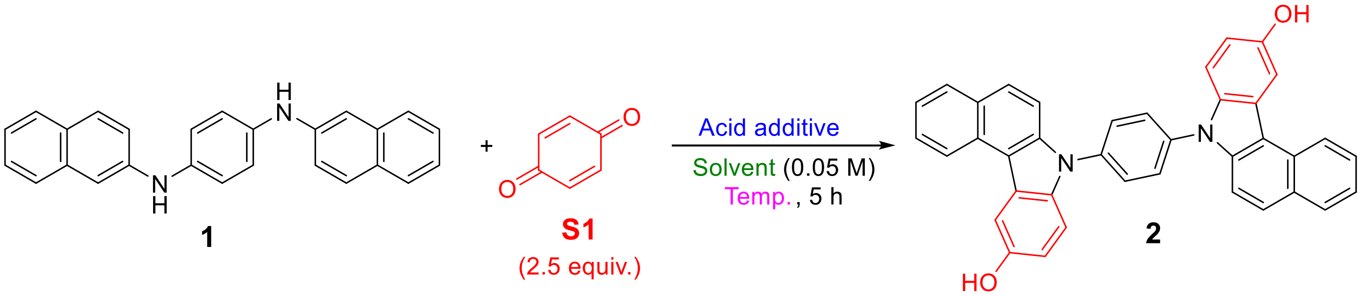

Entry	Acid additive	Solvent	Temp. (°C)	Yield (%)[1]
1	Acetic acid (2.0 equiv.)	Toluene	35	N.D.[2]
2	H_3PO_4 (2.0 equiv.)	Toluene	35	46
3	H_3PO_4 (2.0 equiv.)	DCM	35	35
4	H_3PO_4 (2.0 equiv.)	Toluene	25	40
5	H_3PO_4 (2.0 equiv.)	Toluene	50	56 (54)[3]

[1] Determined by ^{1}H NMR spectroscopy using 1,3,5-trimethoxybenzene as an internal standard. [2] Not detected. [3] Isolated yield.

Table S2. Optimization of the electrochemical sequential reaction of the double carbazole **2** and 2-naphthol

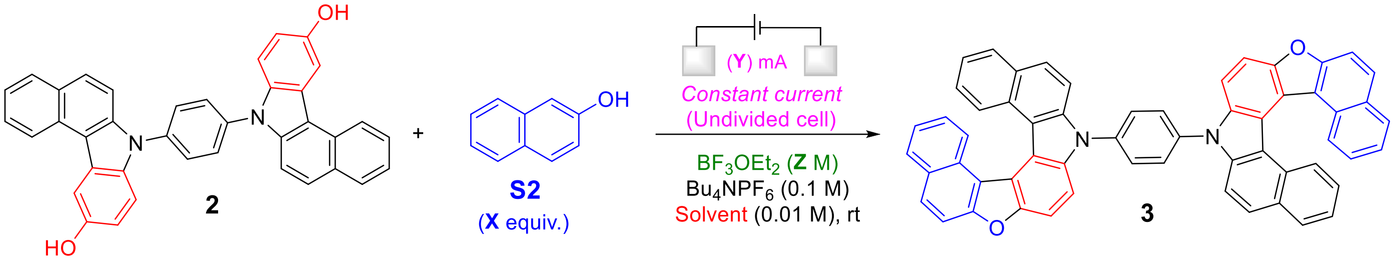

Entry	Anode	Cathode	Current (mA)	S2 (X equiv.)	BF_3OEt_2 (M)	Solvent	Time (h)	Yield (%)[1]
1	C	C	3.0	6.0	0.2	DCM	1.5	14
2	Ni	Ni	3.0	6.0	0.2	DCM	2.5	4
3	Cu	Cu	3.0	6.0	0.2	DCM	1.5	3
4	Pt	Pt	3.0	6.0	0.2	DCM	1.75	19
5	Pt	C	3.0	6.0	0.2	DCM	2.0	10
6	C	Pt	3.0	6.0	0.2	DCM	2.0	8
7	Pt	Pt	3.0	5.0	0.2	DCM	1.75	20
8	Pt	Pt	3.0	4.0	0.2	DCM	1.75	19
9	Pt	Pt	3.0	3.0	0.2	DCM	1.75	13
10	Pt	Pt	2.0	4.0	0.2	DCM	2.5	22
11	Pt	Pt	1.5	4.0	0.2	DCM	3.0	25
12	Pt	Pt	1.0	4.0	0.2	DCM	4.0	24
13	Pt	Pt	1.5	4.0	0.1	DCM	3.5	29 (26)[3]
14	Pt	Pt	1.5	4.0	0.05	DCM	4.5	17
15	Pt	Pt	1.5	4.0	0.1	MeCN	3.0	3
16	Pt	Pt	1.5	4.0	0.1	THF	5.0	N.D.[2]

[1] Determined by ^{1}H NMR spectroscopy using 1,3,5-trimethoxybenzene as an internal standard. [2] Not detected. [3] Isolated yield.

3. A plausible mechanism for the electrochemical domino reaction

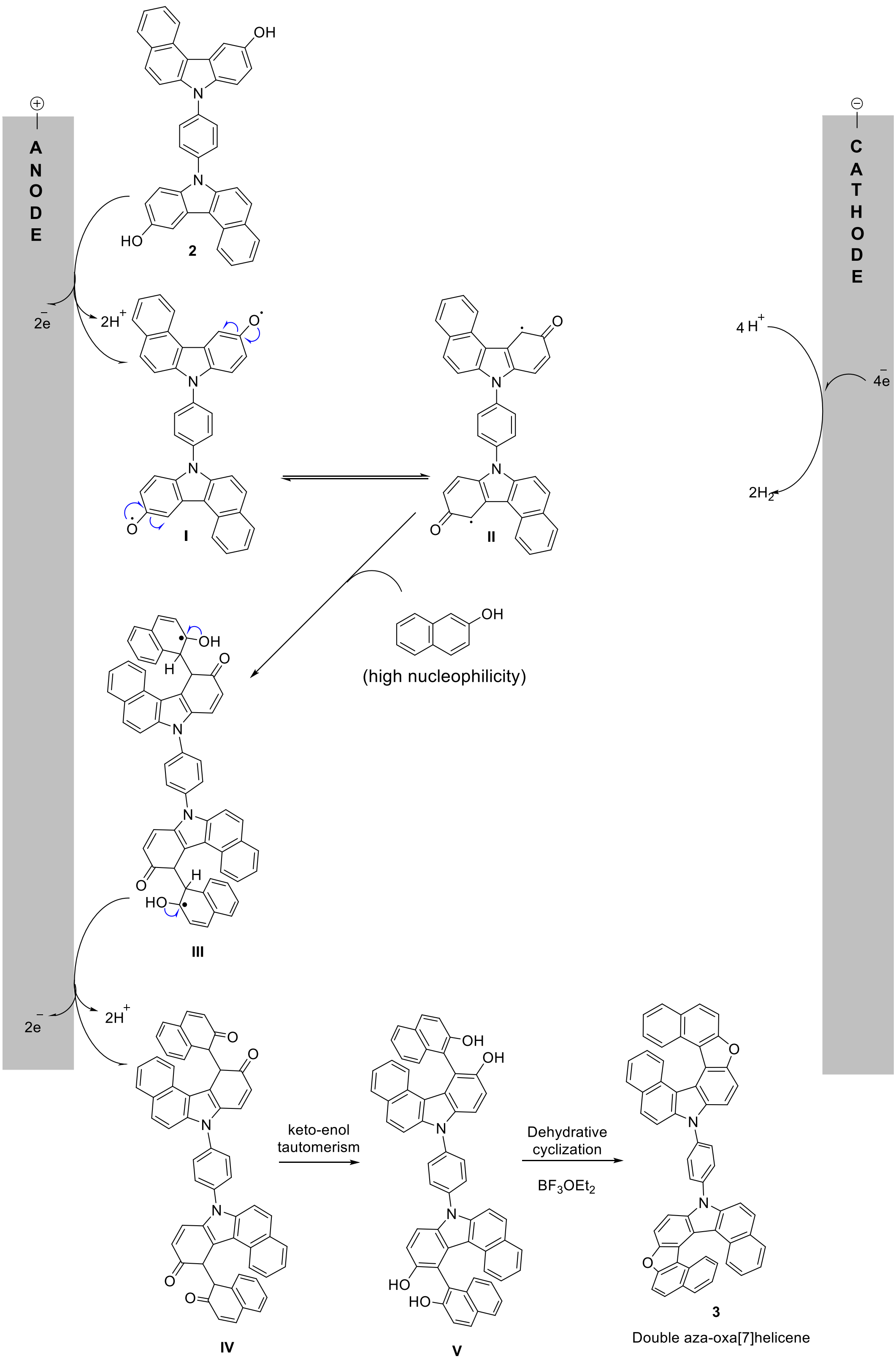

Scheme **S1**

4. HPLC chromatogram of compound 3

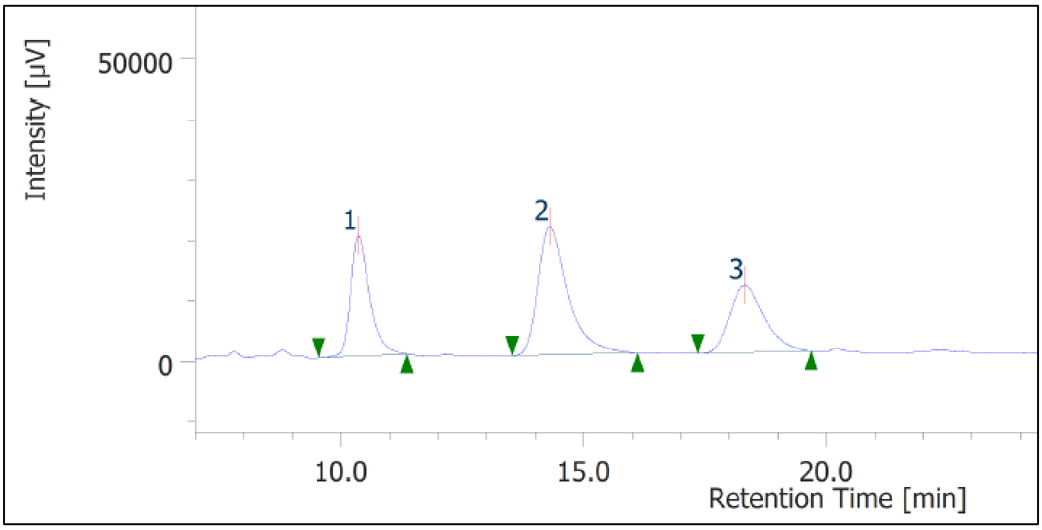

#	Peak Name	CH	tR [min]	Area [µV·sec]	Height [µV]	Area%	Height%	Quantity	NTP	Resolution	Symmetry Factor	Warning
1	Unknown	10	10.363	541472	20008	27.029	38.102	N/A	3775	4.574	1.420	
2	Unknown	10	14.303	911406	21312	45.495	40.585	N/A	2955	3.465	1.581	
3	Unknown	10	18.317	550412	11192	27.475	21.313	N/A	3326	N/A	1.305	

Determined by HPLC (Daicel Chiralpak IA, *n*-hexane/*i*-PrOH = 20/1, flow rate 1.0 mL/min, T = 25 °C, 240 nm): t1= 10.36 min, t2 = 14.30 min, and t3 = 18.32 min.

= Can be separated and collected using HPLC (Daicel Chiralpak IA 30 mmØX, 100 mmL, particle size = 20 µm; *n*-hexane/*i*-PrOH = 9/1; flow rate 5.0 mL/min, T = 25 °C, 290 nm): t_1= 50.71 min, t_2 = 75.88 min, and t_3 = 91.77 min.

5. NMR Spectra

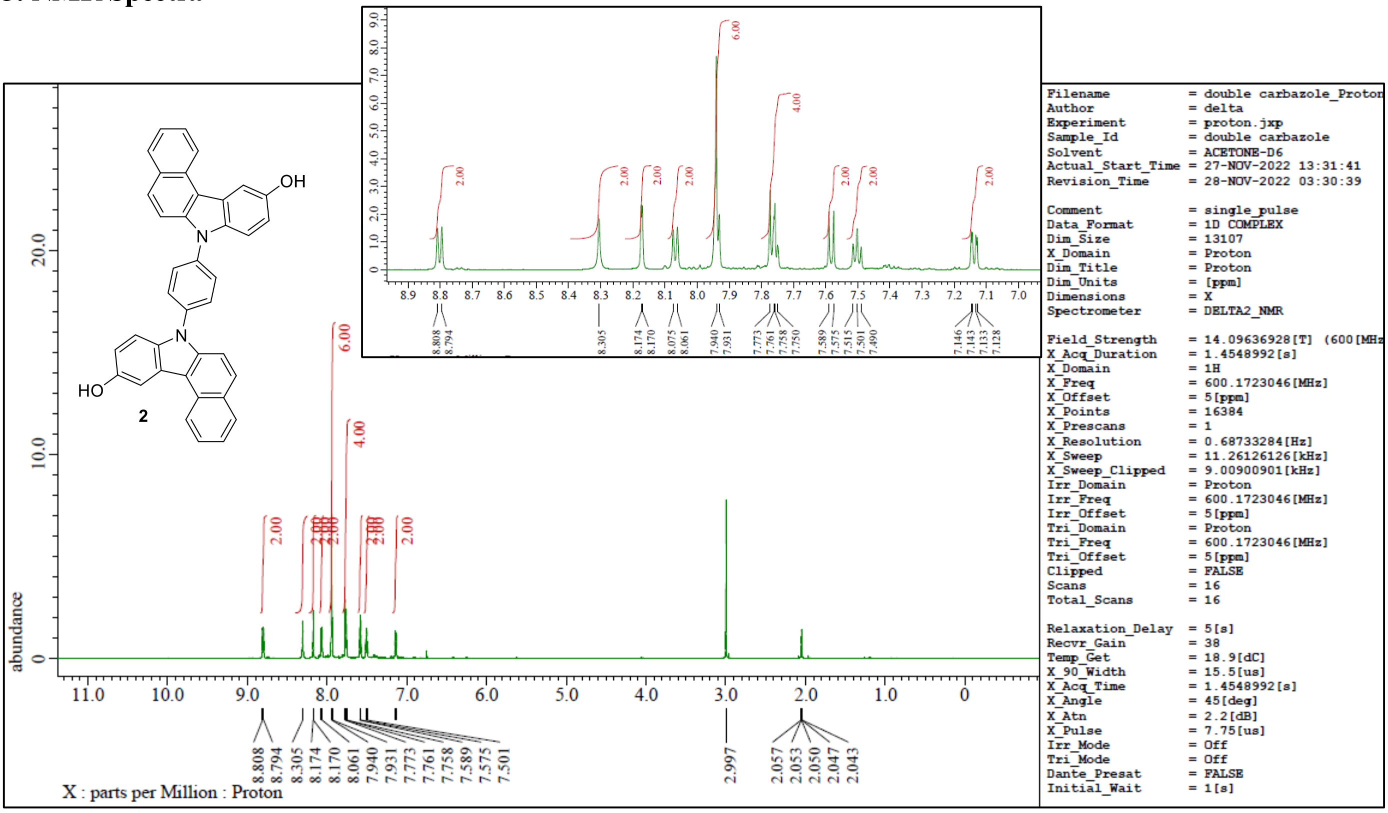

Compound **2** (^{1}H NMR, 600 MHz, $(CD_3)_2CO$).

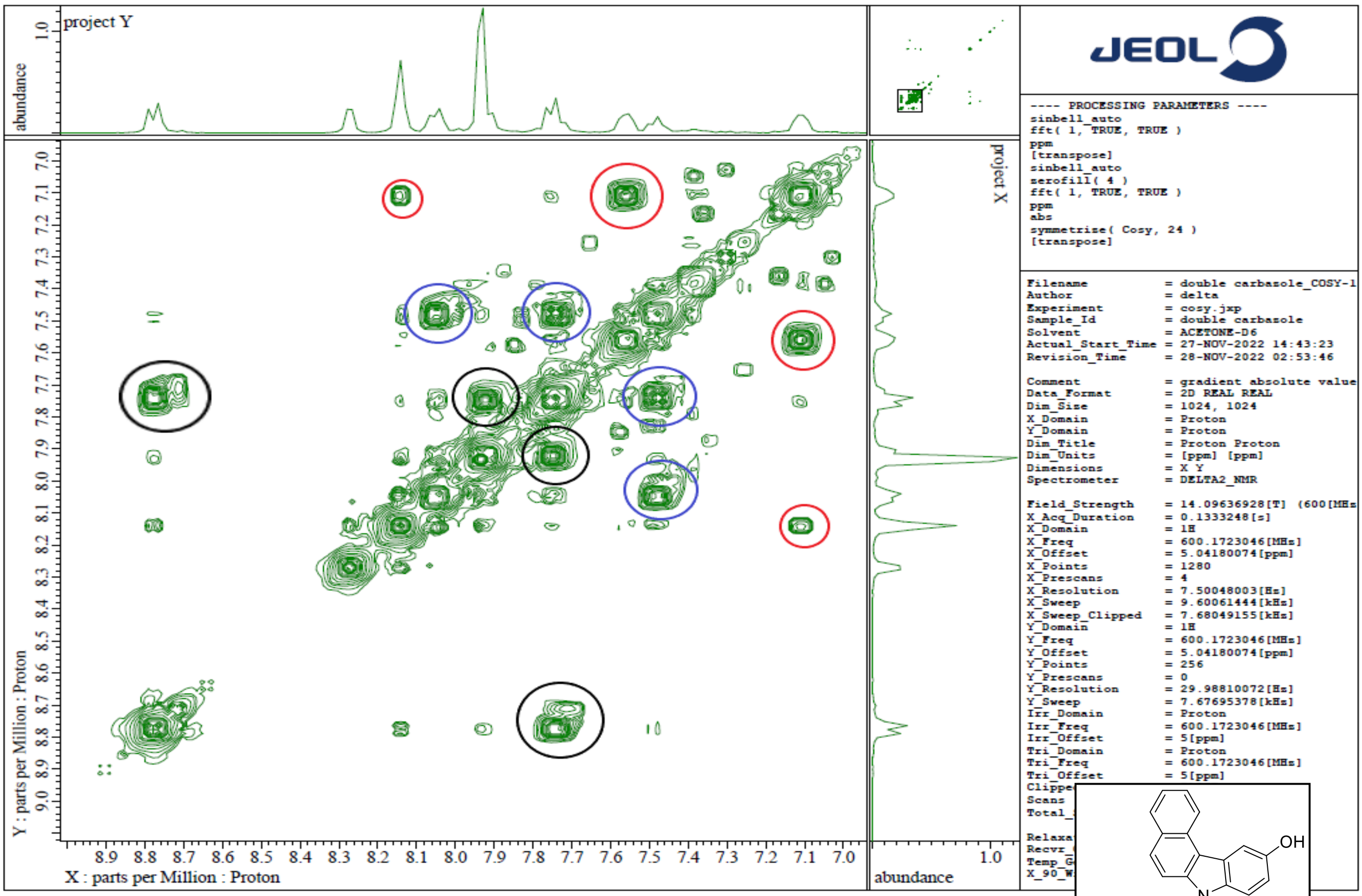

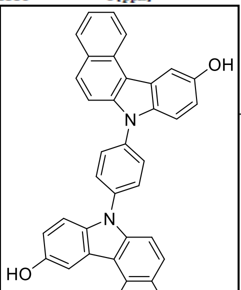

2

Compound **2** (H-H COSY NMR, 600 MHz, $(CD_3)_2CO$).

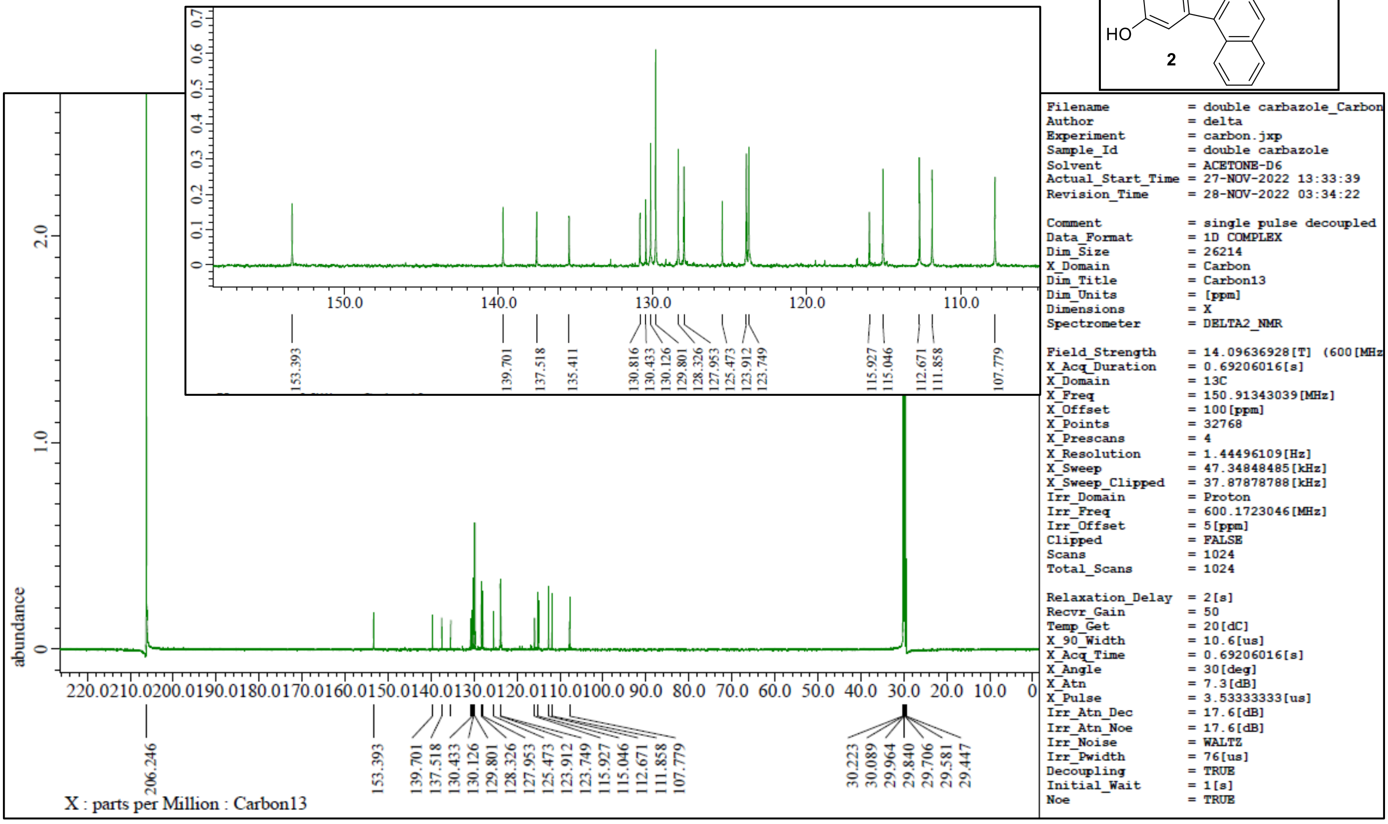

Compound **2** (^{13}C NMR, 151 MHz, $(CD_3)_2CO$).

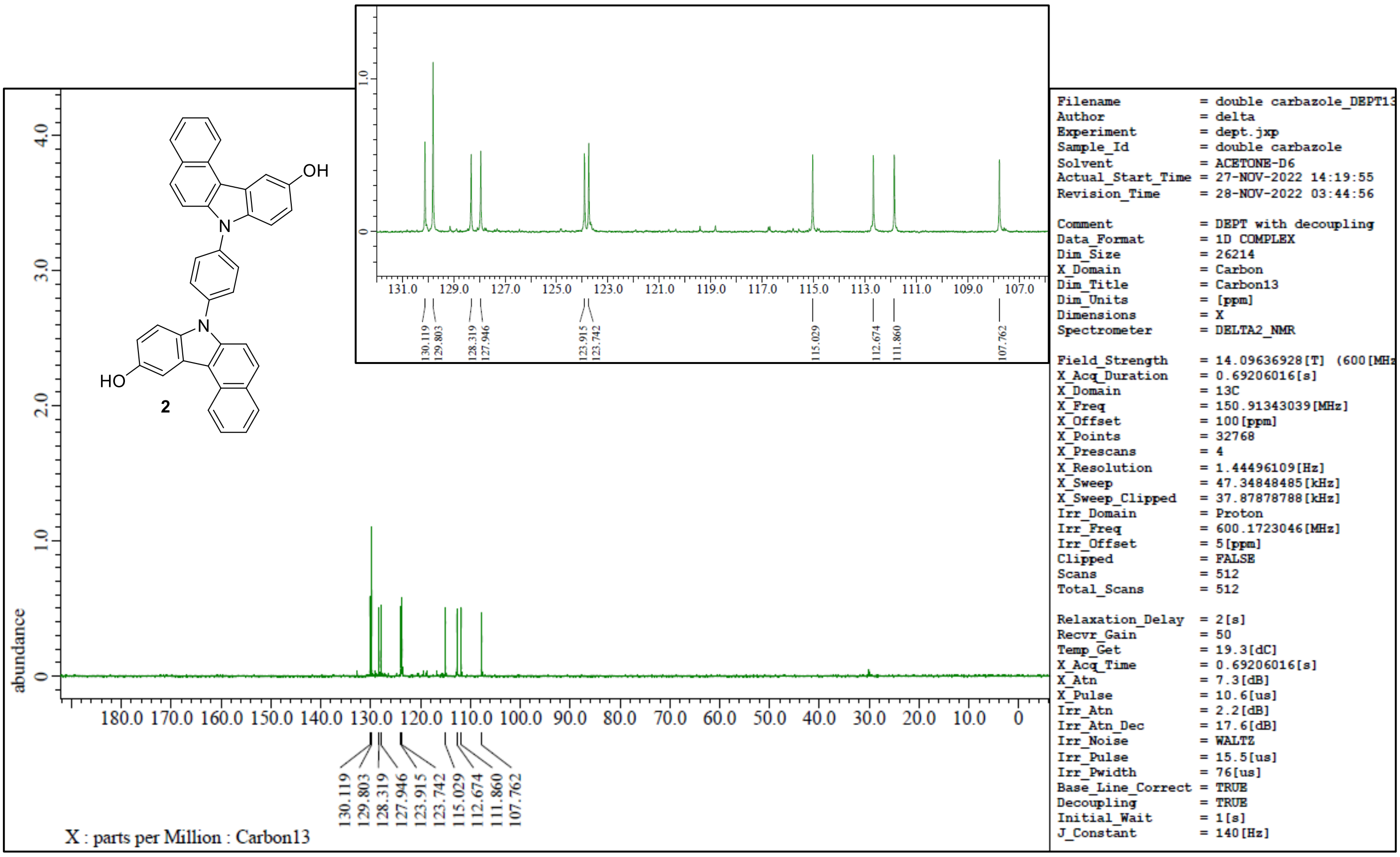

Compound **2** (DEPT-135 NMR, 151 MHz, $(CD_3)_2CO$).

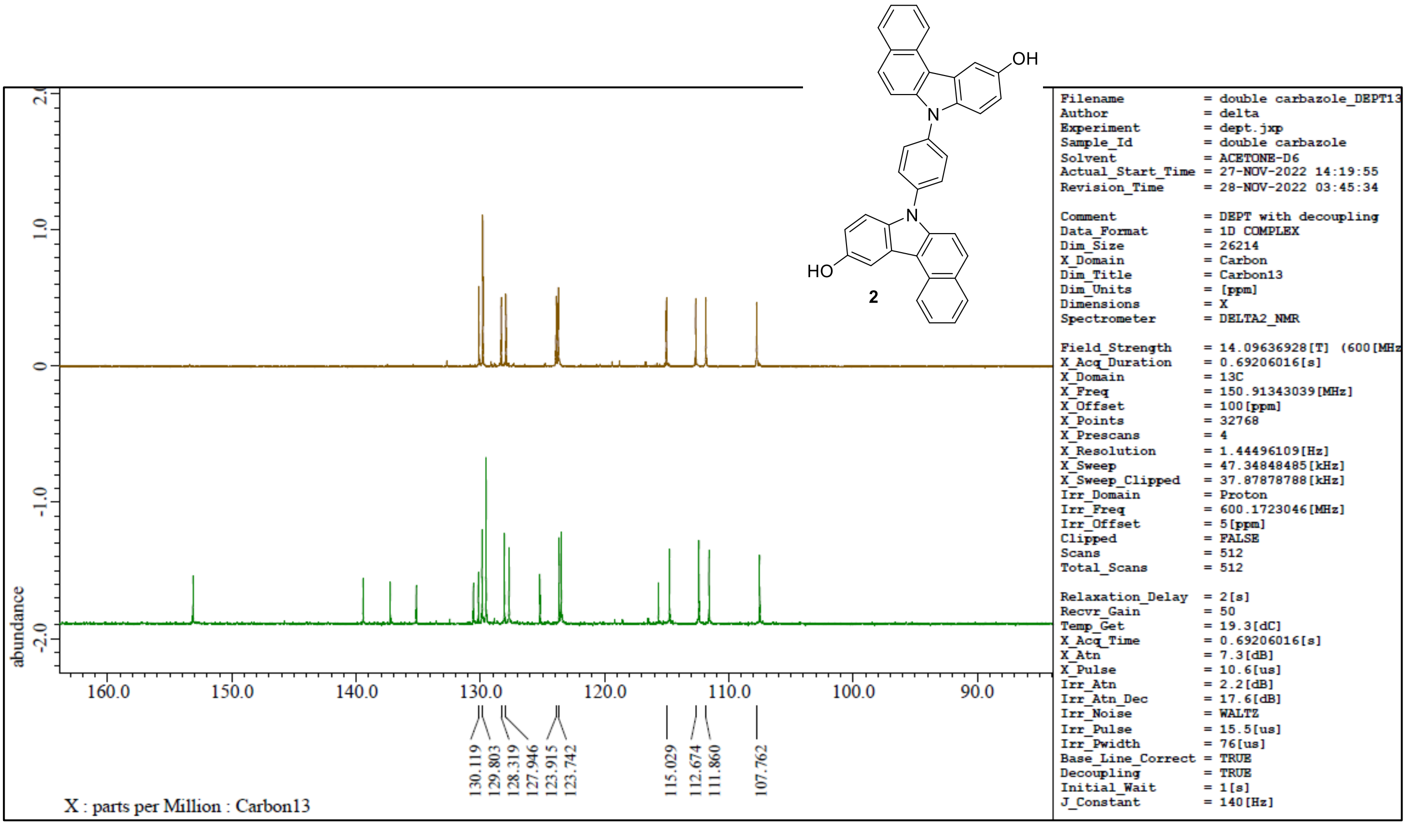

Compound **2** (**DEPT-135 NMR** + **^{13}C NMR**, 151 MHz, $(CD_3)_2CO$).

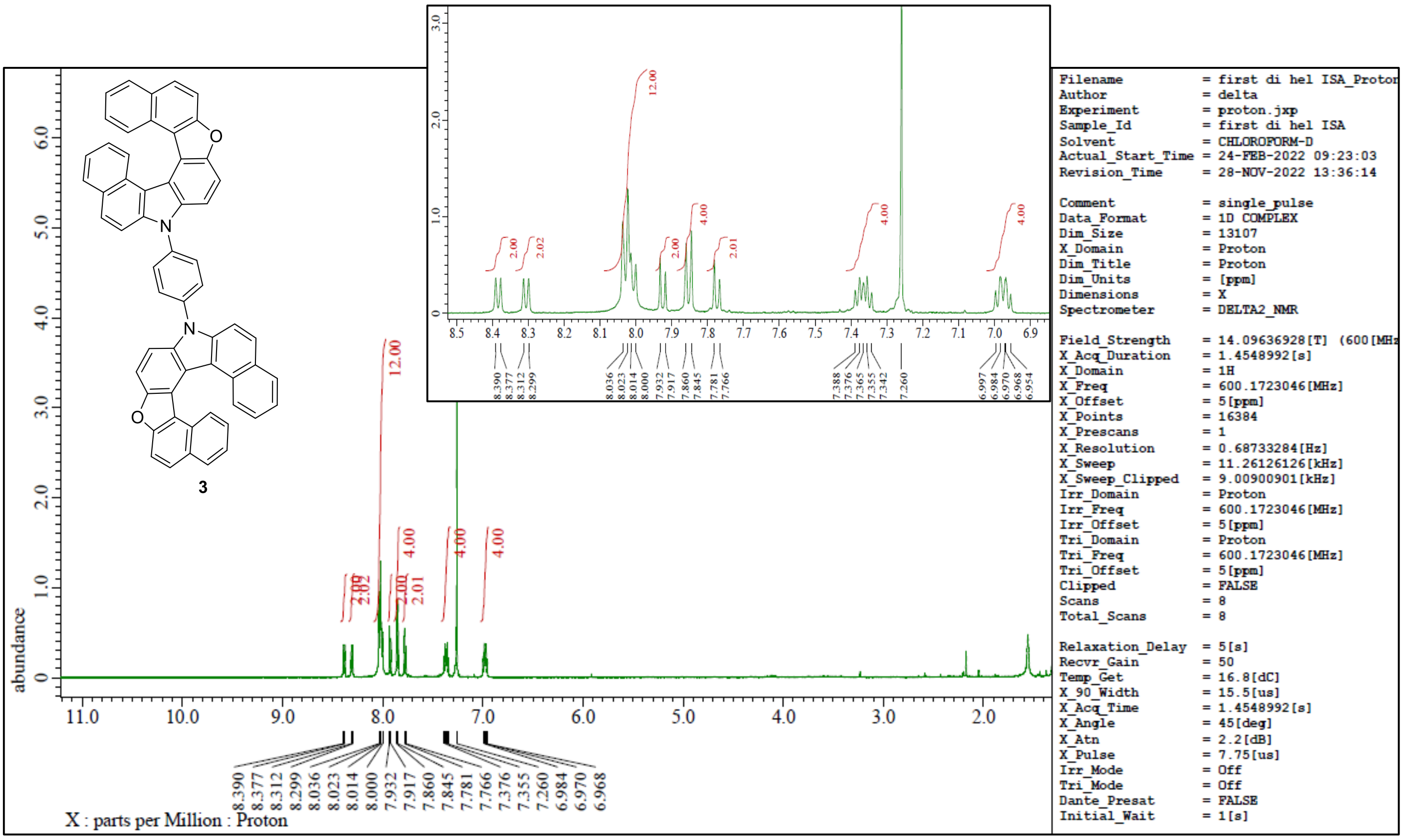

Compound **3** (^{1}H NMR, 600 MHz, $CDCl_3$).

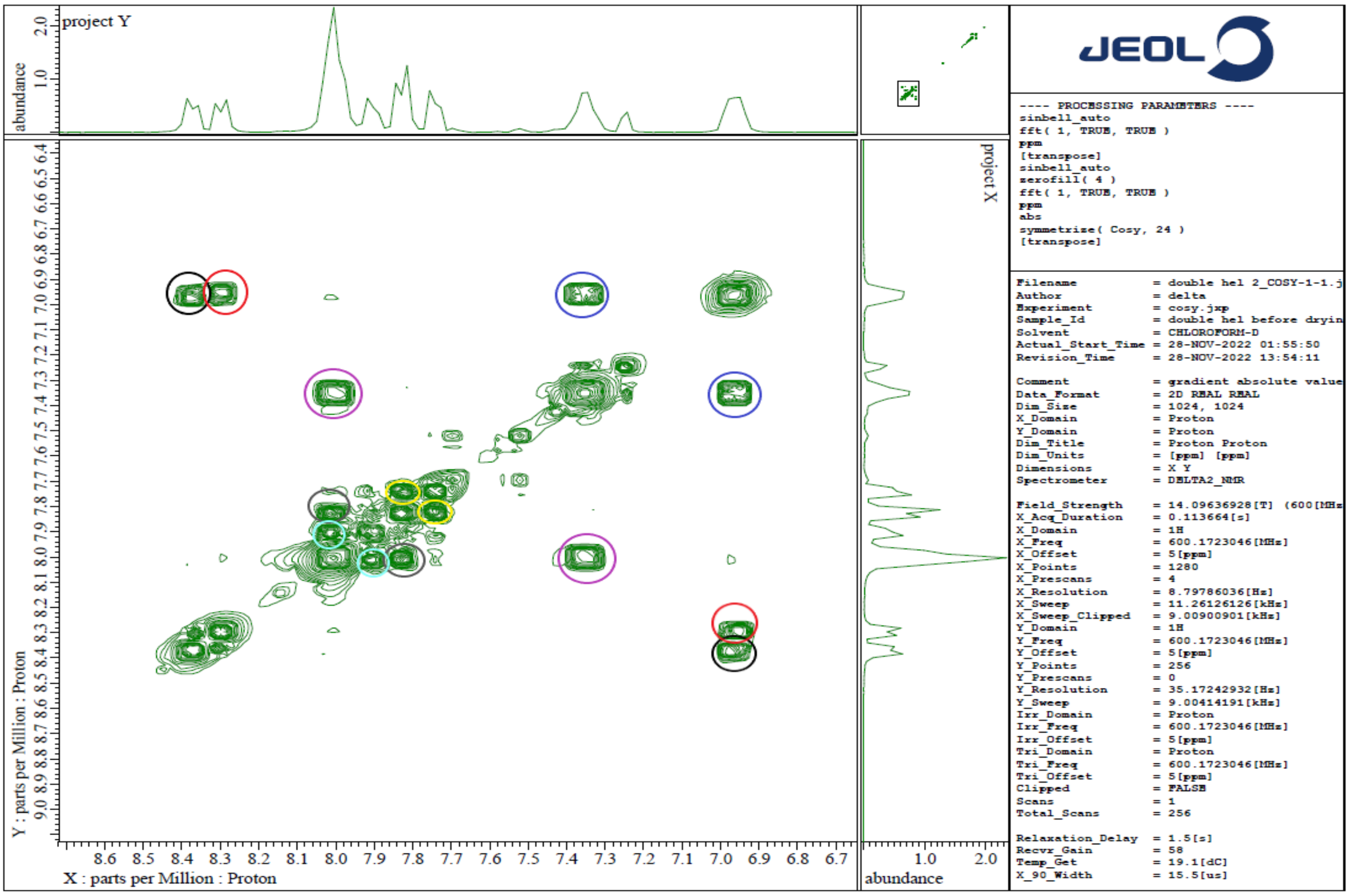

Compound **3** (H-H COSY NMR, 600 MHz, $CDCl_3$).

Based on this H-H COSY, we can assign the ^{1}H NMR as follow:

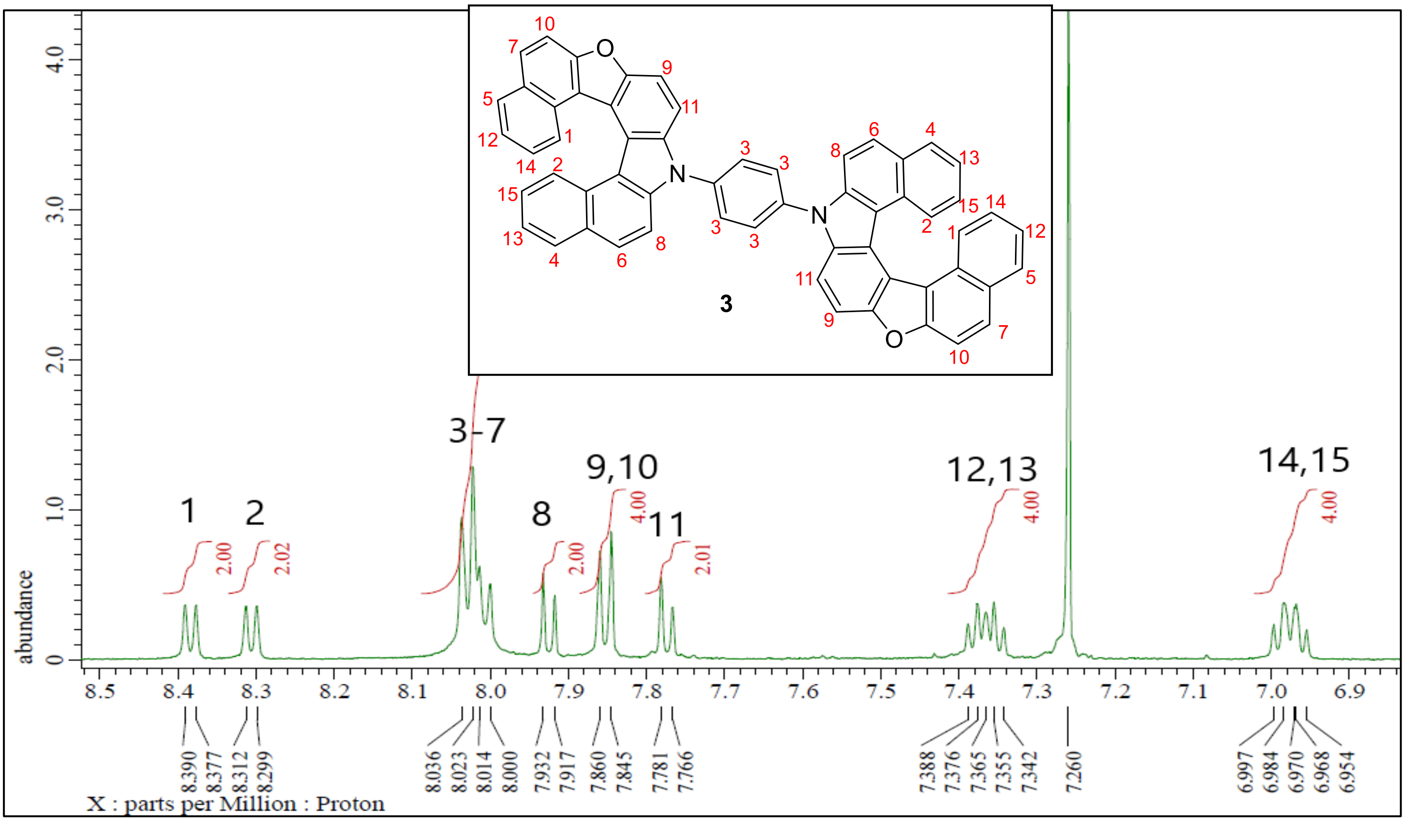

Compound **3** (^{1}H NMR, 600 MHz, CDCl$_3$).

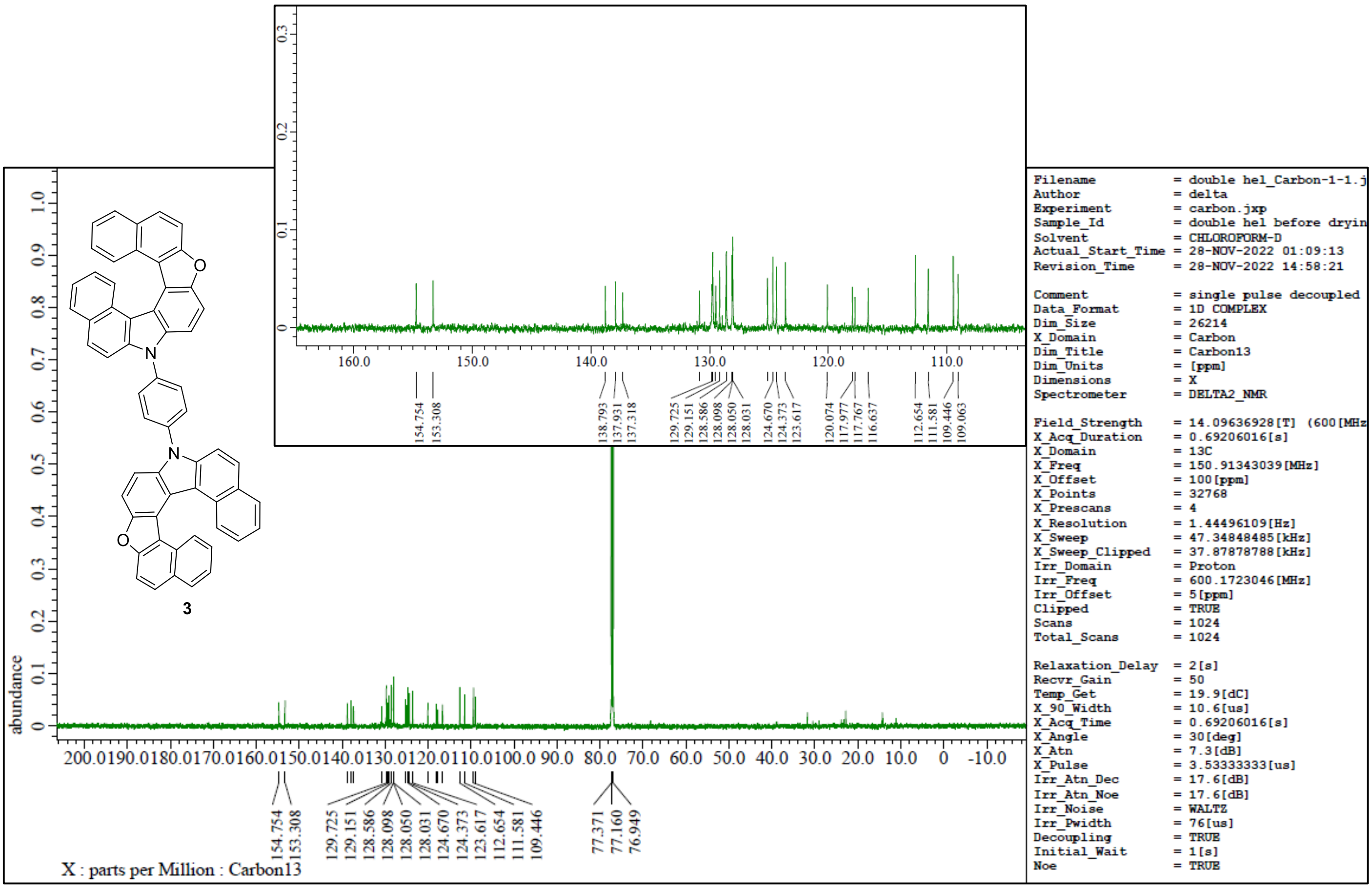

Compound **3** (^{13}C NMR, 151 MHz, CDCl$_3$).

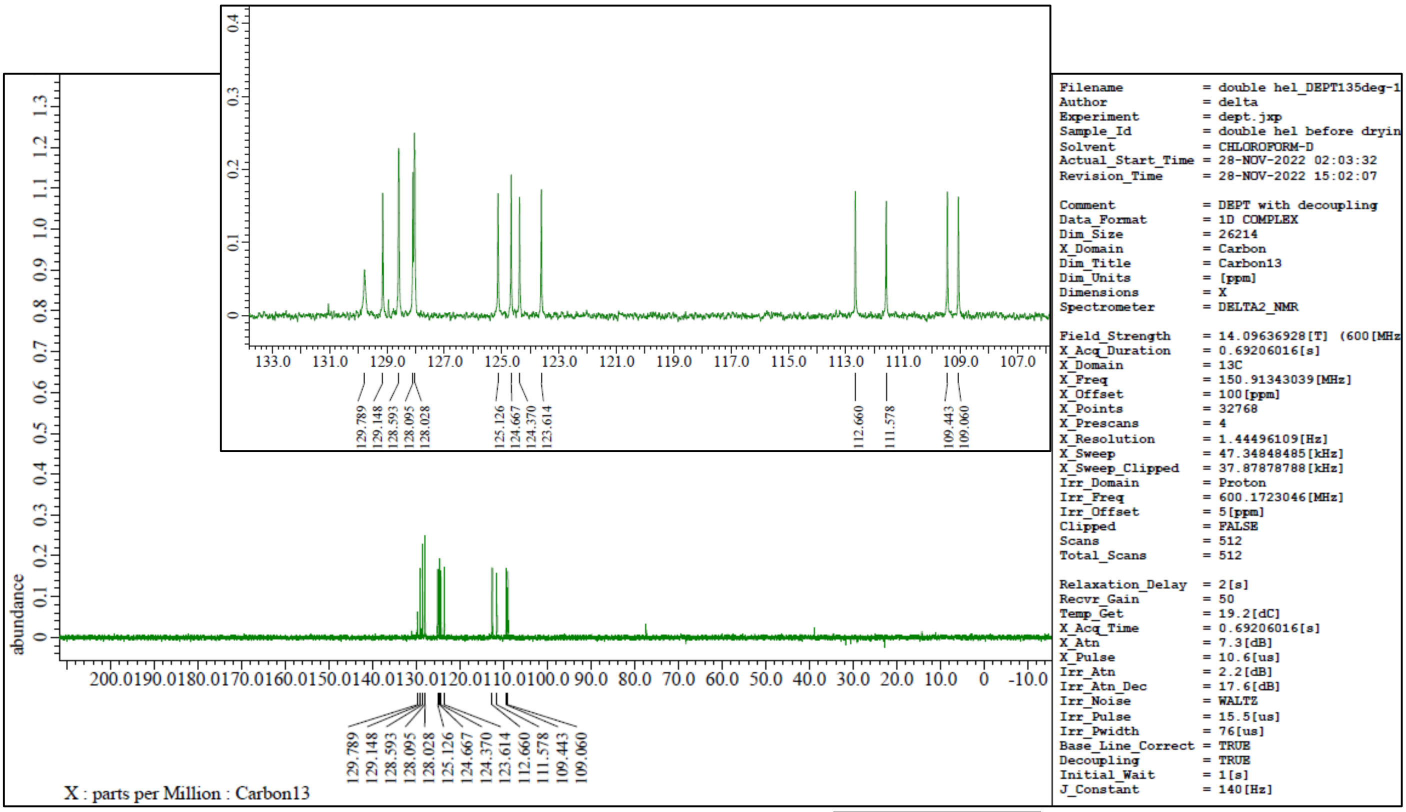

Compound **3** (DEPT-135 NMR, 151 MHz, $CDCl_3$).

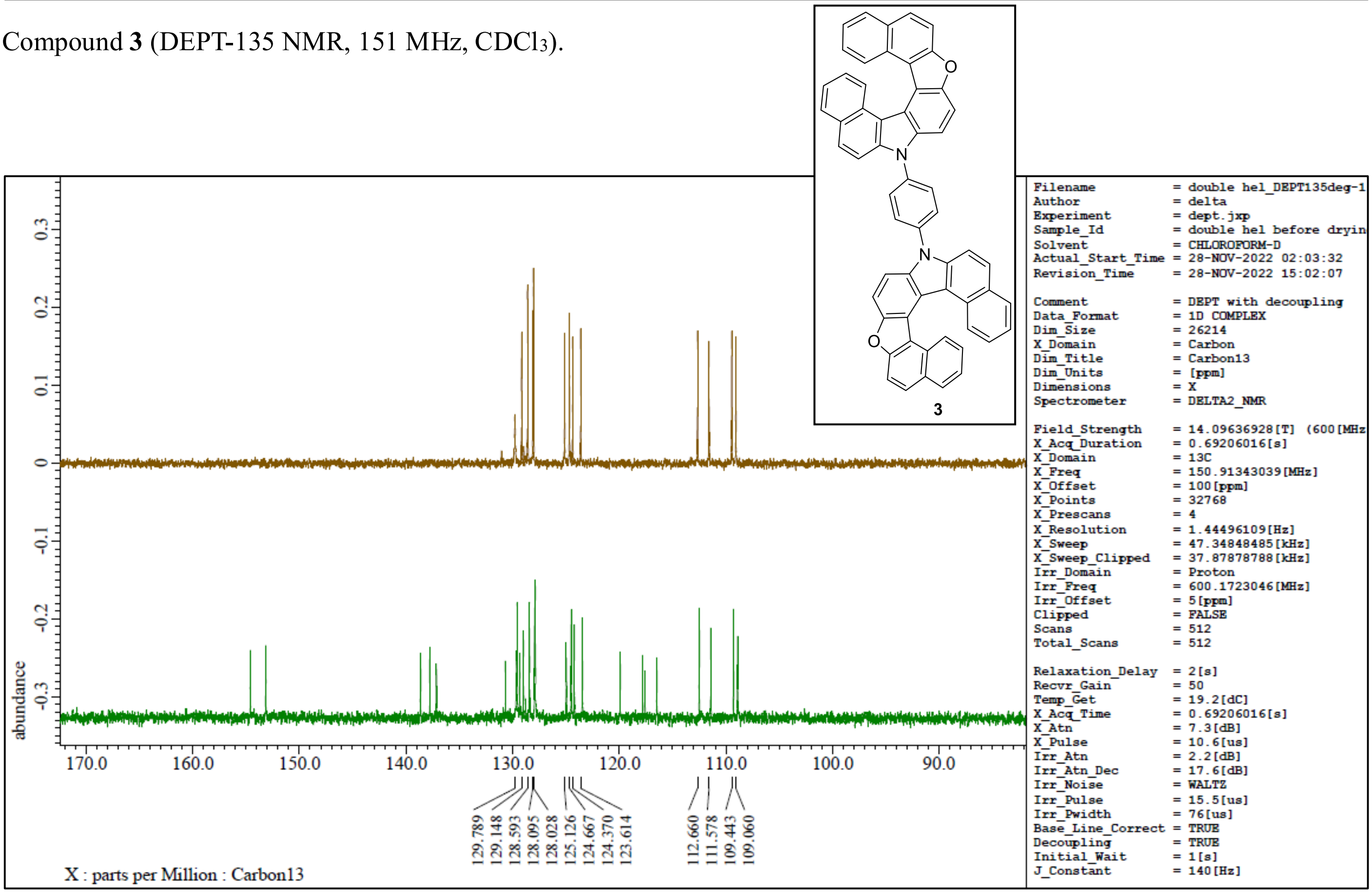

Compound **3** (**DEPT-135 NMR** + **^{13}C NMR**, 151 MHz, $CDCl_3$).

6. Epimerization barrier study of 3

Since epimerization is a first-order process, we could monitor the change in the diasteromeric excess ratio (de%) with time at three different temperatures of 40 - 60 °C after separating the first peak t_1= 10.36 min, and monitor its epimerization to the meso isomer (middle peak, t_2 = 14.30 min).

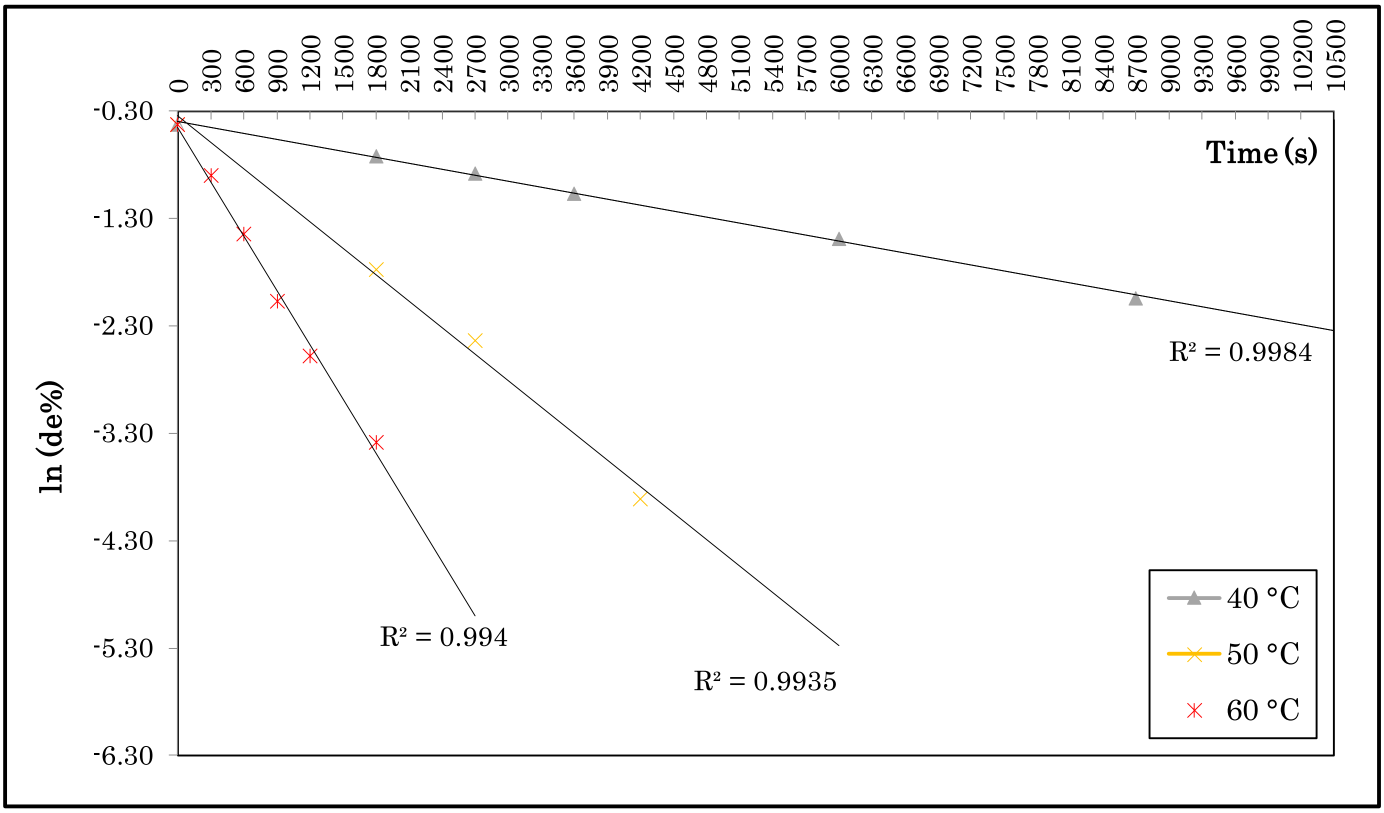

A plot showing ln(de) versus time in seconds to show the epimerization rate of **3** as it was heated at 40, 50, 60 °C, at 1 mg/mL concentration in toluene

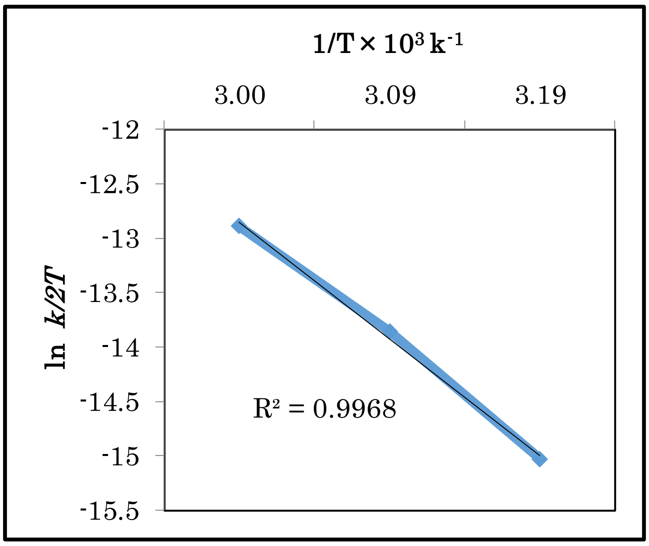

Eyring plot of **3** showing the change in ln(k/2T) versus 1/T

$y = -11185.820 \cdot x + 20.713$

$\Delta H^{\neq} = 93.005$ kJ.mol^{-1}

$\Delta S^{\neq} = -25.325$ J.mol^{-1} K^{-1}

$\Delta G^{\neq} = \Delta H^{\neq} - T\,\Delta S^{\neq}$

$\Delta G^{\neq} = 101.188 \pm 0.253$ kJ.mol^{-1}

$\Delta G^{\neq}$ at 40 °C = 100.935 kJ.mol^{-1}

$\Delta G^{\neq}$ at 50 °C = 101.187 kJ.mol^{-1}

$\Delta G^{\neq}$ at 60 °C = 101.442 kJ.mol^{-1}

$t_{1/2}$ at 25 °C = 6.41 h

$t_{1/2}$ at -20 °C = 248 days

7. DFT Calculations

Theoretical calculations were performed by the density functional theory (DFT) method using the Gaussian 16 software package [1]. The geometries of (*P*,*M*)-**3** were optimized at three different levels of theory; B3LYP/6-311G(d,p), wB97XD/6-311G(d,p), and MN15/6-311G(d,p) [2] and compared to the geometrics obtained from X-ray crystallographic analysis. The time-dependent density functional theory (TD-DFT) calculation was conducted at the MN15/6-311G(d,p) and B3LYP/6-311G(d,p) levels after the geometry optimization at the B3LYP/6-311G(d,p) level [3,4]. IRC calculations were performed to check the transition states and study the isomerization barriers. Nucleus-independent chemical shifts (NICS) were evaluated by using the gauge invariant atomic orbital (GIAO) approach at the GIAO-B3LYP/6-311+G(2d,p) and GIAO-MN15/6-311+G(2d,p) levels [5-7].

Table S3. Selected experimental and calculated structural parameters of double aza-oxa[7]helicene **3**.

Parameters	Experimental	B3LYP[1]	wB97XD[1]	MN15[1]
Centroids' distance (rings F'-H')	4.949 A°	4.885 A°	4.721 A°	4.759 A°
d_1-N_7-d_2 Centroid angle	46.36°	45.43°	44.24°	45.51°
C_5-C_6-N_7-C_9 Dihedral angle	54.38°	60.25°	59.03°	54.72°
C_1-C_6-N_7-C_8 Dihedral angle	41.86°	57.08°	55.34°	51.42°
C_1-C_{15} Distance	3.181A°	3.316 A°	3.281 A°	3.241 A°
C_5-C_{14} Distance	3.166 A°	3.356 A°	3.316 A°	3.266 A°
C_{10}-C_{11} Distance	3.040 A°	3.071 A°	2.986 A°	3.001 A°
C_{12}-C_{13} Distance	4.158 A°	4.135 A°	3.697 A°	3.998 A°

[1] All calculations are carried out using 6-311G(d,P) basis set at three different functions (B3LYP, wB97XD, and MN15).

Figure S1: crystal measurements:

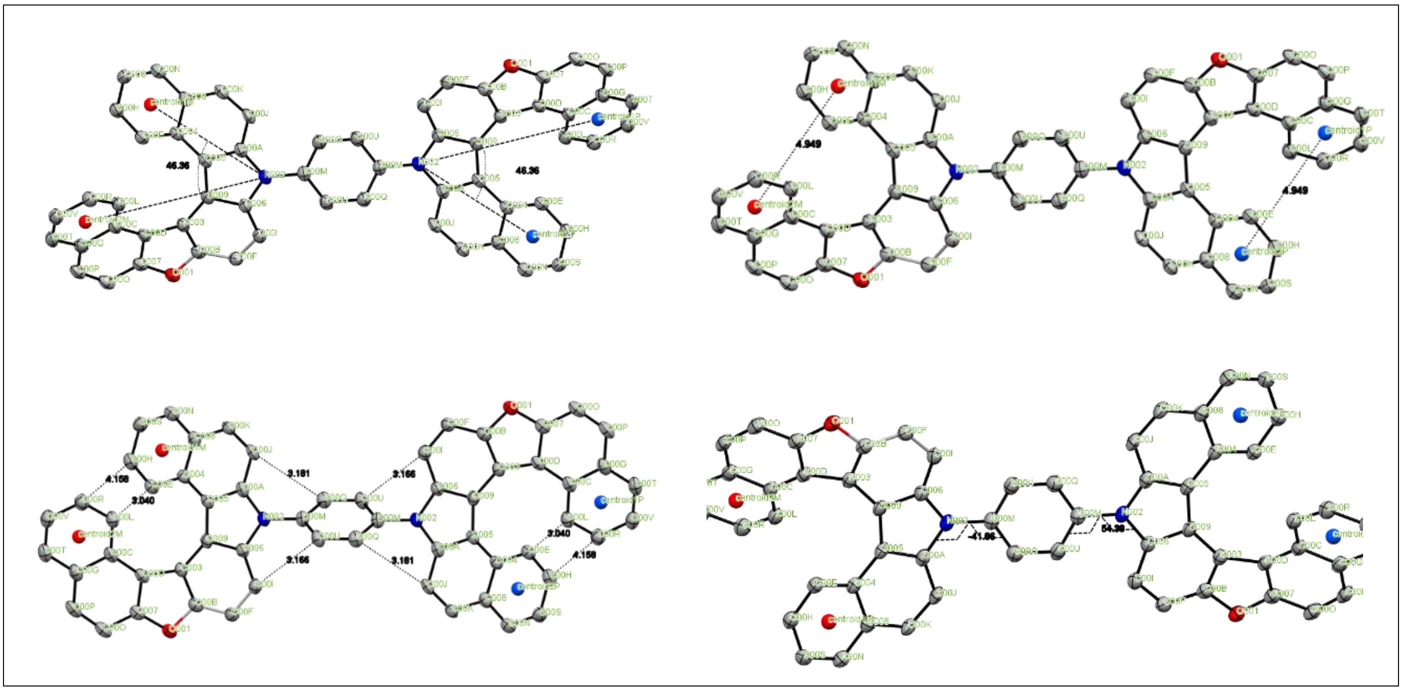

Figure S2: measurements of the optimized structures:

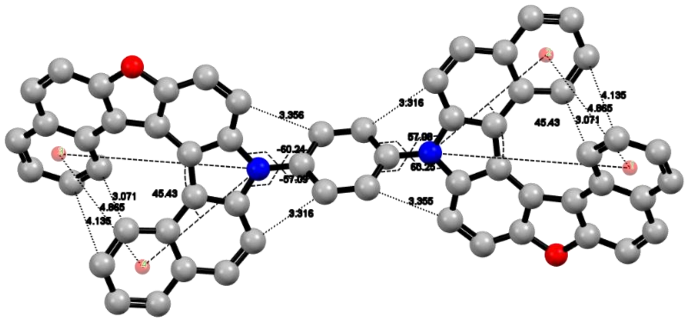

Optimized structure at B3LYP/6-311G(d,p) level

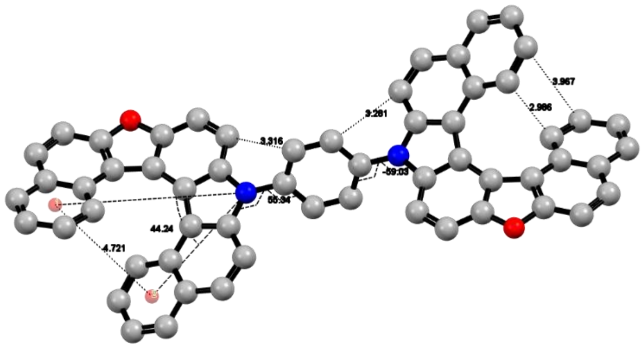

Optimized structure at wB97XD/6-311G(d,p) level

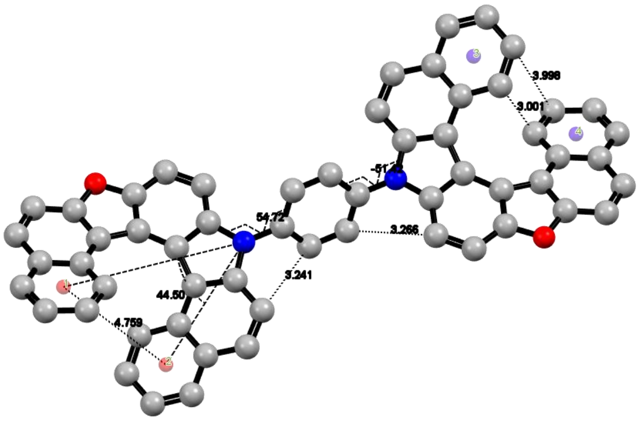

Optimized structure at MN15/6-311G(d,p) level of theory

Figure S3: Selected molecular orbitals of **3** (isoval = 0.02).

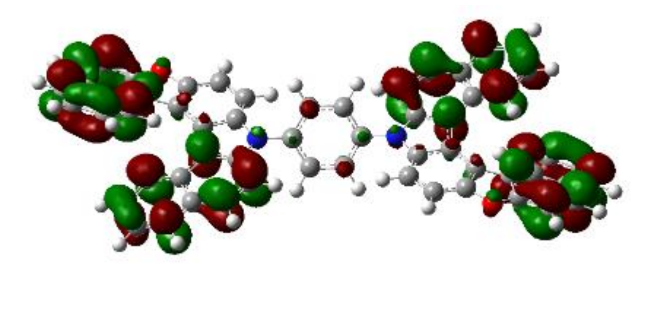	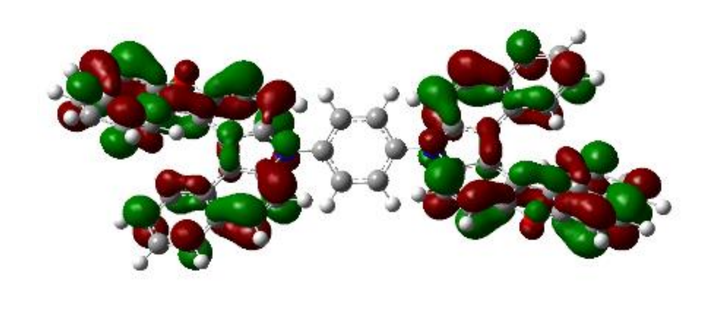
LUMO+2 (-5.20 eV)	LUMO+1 (-5.68 eV)
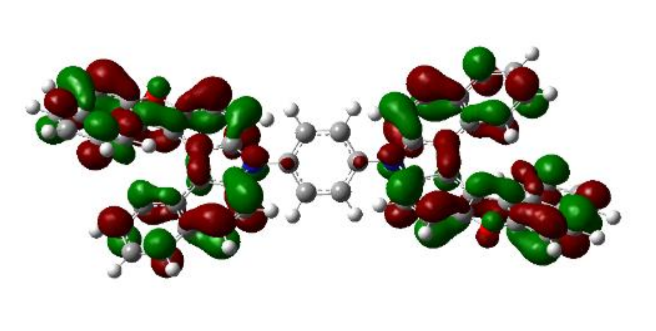	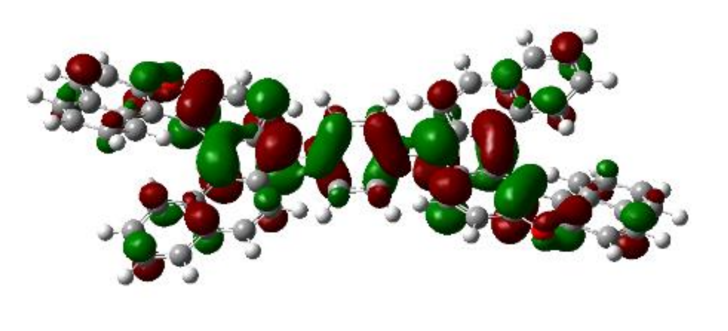
LUMO (-5.72 eV)	HOMO (-7.90 eV)
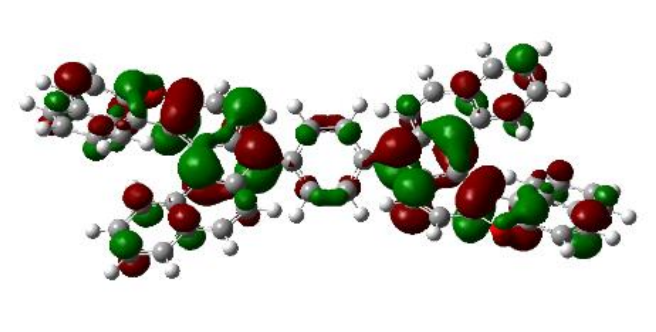	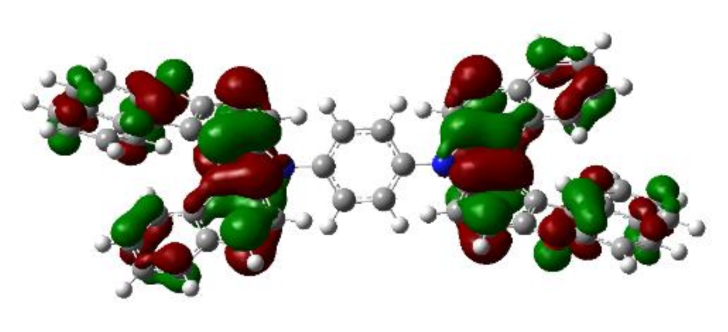
HOMO-1 (-8.11 eV)	HOMO-2 (-8.30 eV)

Table S4. Summary of the TD-DFT calculation results of **3** at B3LYP/6-311G(d,p) in gas phase

Excited states	Energy (eV)	Wavelength (nm)	Oscillator strength	Major contributions		
S_1	3.0862	401.74	0.7280	HOMO	LUMO	(62%)
				HOMO-1	LUMO+1	(32%)
S_2	3.1339	395.62	0.00001	HOMO	LUMO+1	(50%)
				HOMO-1	LUMO	(48%)
S_3	3.2876	377.13	0.00001	HOMO	LUMO+1	(49%)
				HOMO-1	LUMO	(48%)
S_4	3.3062	375.01	0.0550	HOMO-1	LUMO+1	(61%)
				HOMO	LUMO	(31%)
S_5	3.4481	359.57	0.2632	HOMO-2	LUMO	(62%)
				HOMO-3	LUMO+1	(23%)
				HOMO	LUMO	(11%)

S_6	3.4915	355.10	0.0000	HOMO-2 LUMO+1 (56%)
				HOMO-3 LUMO (35%)
				HOMO-1 LUMO+9 (1%)
				HOMO LUMO+8 (1%)
S_7	3.5686	347.43	0.0698	HOMO LUMO+2 (67%)
S_8	3.6268	341.86	0.0000	HOMO-1 LUMO+2 (68%)
S_9	3.6533	339.38	0.0239	HOMO LUMO+3 (68%)
S_{10}	3.6857	336.40	0.0000	HOMO-3 LUMO (57%)
				HOMO-2 LUMO+1 (37%)
				HOMO-1 LUMO+3 (1.3%)

Only S1 ~ S3 have been displayed.

Table S5. Summary of the TD-DFT calculation results of **3** at MN15/6-311G(d,p) in gas phase

**Excited states**	**Energy (eV)**	**Wavelength (nm)**	**Oscillator strength**	**Major contributions**
S_1	3.3837	366.41	0.8669	HOMO LUMO (52%)
				HOMO-1 LUMO+1 (44%)
S_2	3.4170	362.84	0.00001	HOMO LUMO+1 (49%)
				HOMO-1 LUMO (48%)
S_3	3.7639	329.41	0.4107	HOMO-2 LUMO (51%)
				HOMO-3 LUMO+1 (36%)
				HOMO-1 LUMO+1 (10%)

Only S1 ~ S3 have been displayed.

Table S6. Summary of the TD-DFT calculation results of **3** at MN15/6-311G(d,p) in chloroform

**Excited states**	**Energy (eV)**	**Wavelength (nm)**	**Oscillator strength**	**Major contributions**
S_1	3.3434	370.84	1.0617	HOMO LUMO (52%)
				HOMO-1 LUMO+1 (45%)
S_2	3.3707	367.82	0.00001	HOMO LUMO+1 (49%)
				HOMO-1 LUMO (48%)
S_3	3.7361	331.86	0.6018	HOMO-2 LUMO (51%)
				HOMO-3 LUMO+1 (37%)
				HOMO-1 LUMO+1 (10%)
				HOMO-1 LUMO+8 (1%)
				HOMO LUMO+9 (1%)
S_4	3.7700	328.87	0.00001	HOMO-2 LUMO+1 (48%)
				HOMO-3 LUMO (41%)
S_5	4.1102	301.65	0.0953	HOMO LUMO+2 (43%)
				HOMO-4 LUMO (25%)
				HOMO-5 LUMO+1 (18%)

Only S1 ~ S5 have been displayed.

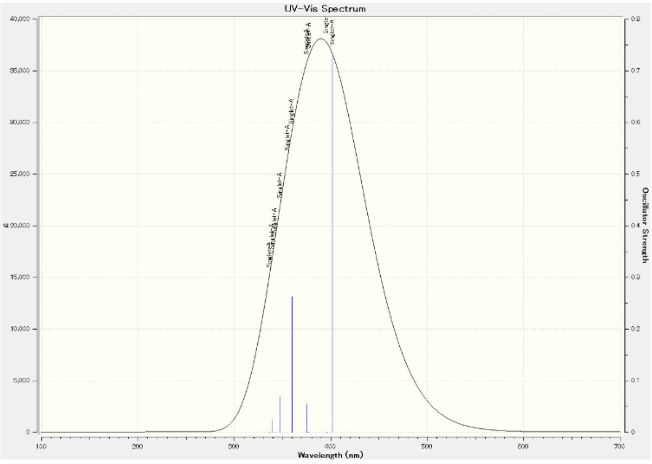

Figure S4a: Simulated UV-vis absorption spectra of (*P*,*M*)-**3** at B3LYP/6-311G(d,p) in the gas phase; (Wavelength (nm) = 401.74, Oscillator Strength = 0.728).

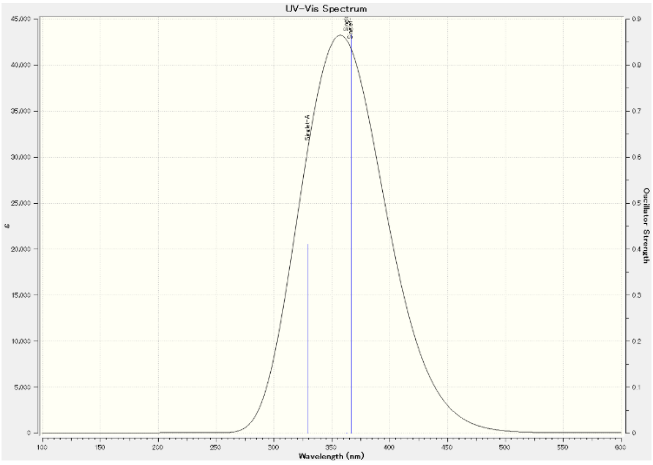

Figure S4b: Simulated UV-vis absorption spectra of (*P*,*M*)-**3** at MN15/6-311G(d,p) in the gas phase; (Wavelength (nm) = 366.41, Oscillator Strength = 0.867).

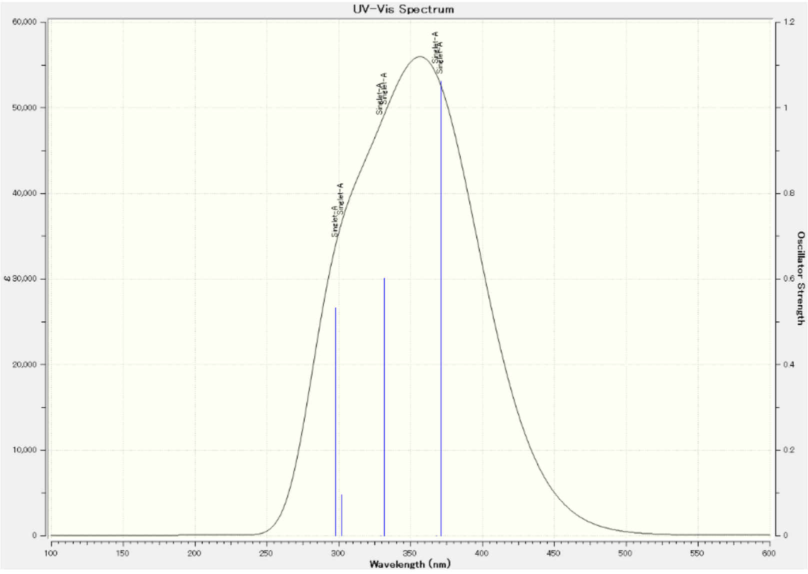

Figure S4c: Simulated UV-vis absorption spectra of (*P*,*M*)-**3** at MN15/6-311G(d,p) in chloroform; (Wavelength (nm) = 370.84, Oscillator Strength = 1.062).

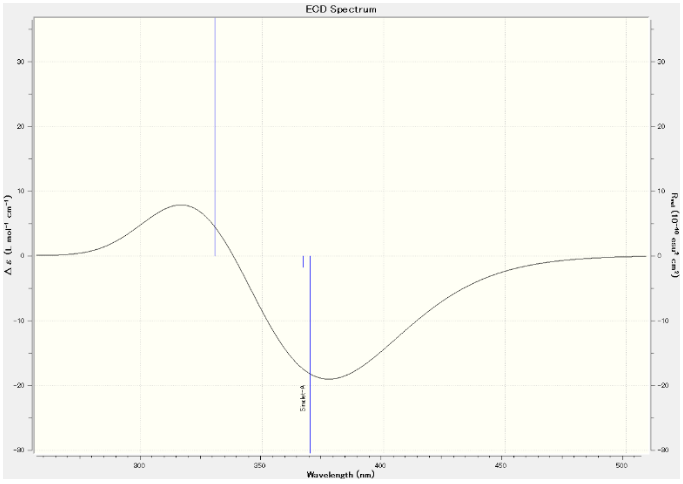

Figure S4d: Simulated ECD spectrum of (*M*,*M*)-**3** at MN15/6-311G(d,p) in the gas phase; (Wavelength (nm) = 369.79, R_{vel} (10^{-40} esu^2 cm^2) = -110.5678.

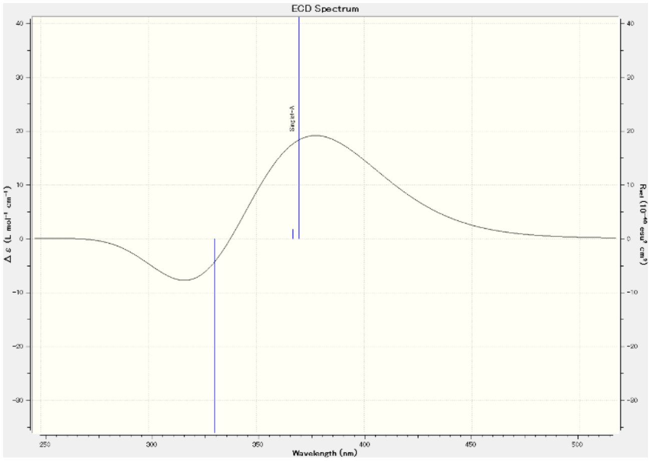

Figure S4e: Simulated ECD spectrum of (*P*,*P*)-**3** at MN15/6-311G(d,p) in the gas phase; (Wavelength (nm) = 369.79, R_{vel} (10^{-40} esu^2 cm^2) = 110.5655.

Figure S5: NICS(0) calculations

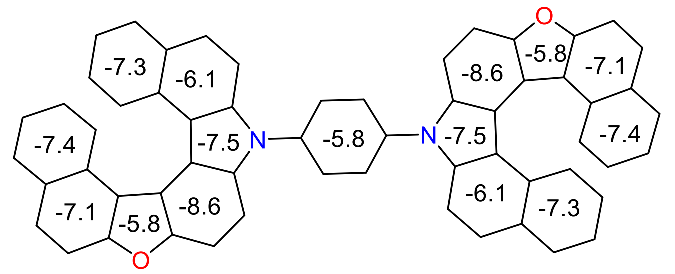

Figure S5a: NICS(0) values of (*P*,*M*)-**3** calculated at MN15/6-311+G(2d,p) level of theory.

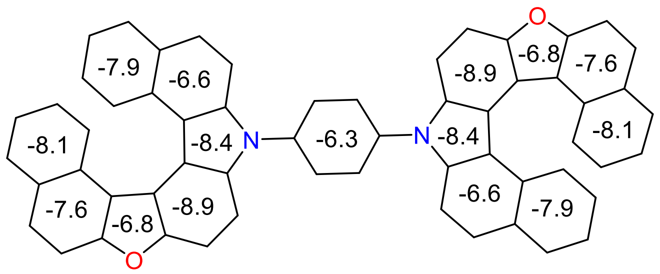

Figure S5b: NICS(0) values of (*P*,*M*)-**3** calculated at B3LYP/6-311+G(2d,p) level of theory.

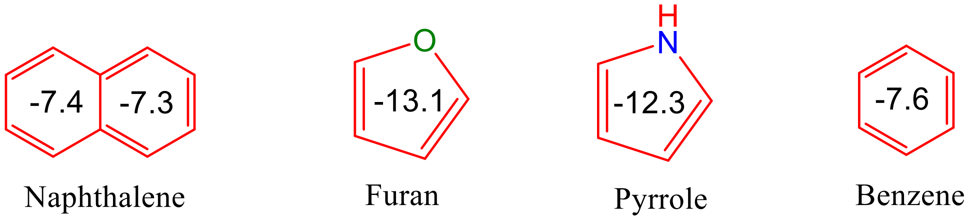

Figure S5c: NICS(0) values of some aromatic rings calculated at MN15/6-311+G(2d,p) level of theory.

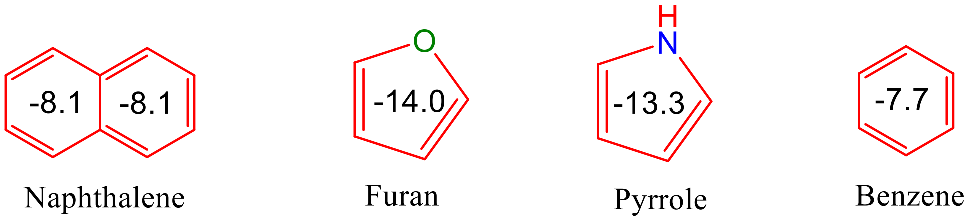

Figure S5c: NICS(0) values of some aromatic rings calculated at B3LYP/6-311+G(2d,p) level of theory.

Cartesian coordinates

(*P*,*M*)-**3** optimized at B3LYP/6-311G(d,p) in the gas phase; *E* = -2490.04224 Hartree

1	C	-1.392399	-0.096100	-0.087851
2	C	-0.581329	-0.791991	-0.988408
3	C	0.802823	-0.697070	-0.900056
4	C	1.392403	0.096118	0.087830
5	C	0.581334	0.792010	0.988386
6	C	-0.802818	0.697087	0.900035
7	N	2.807897	0.187385	0.175818
8	N	-2.807892	-0.187370	-0.175838
9	C	3.677485	-0.881903	0.399021
10	C	5.014944	-0.395902	0.380365

11	C	4.918560	1.048547	0.317253
12	C	3.557991	1.353782	0.121865
13	C	-3.677485	0.881919	-0.399019
14	C	-5.014943	0.395913	-0.380363
15	C	-4.918553	-1.048537	-0.317266
16	C	-3.557981	-1.353770	-0.121895
17	C	5.823237	2.129852	0.585895
18	C	5.347959	3.468111	0.387332
19	C	3.993015	3.695230	0.002617
20	C	3.091936	2.668875	-0.092116
21	C	3.329098	-2.201759	0.716367
22	C	4.341183	-3.103613	1.002327
23	C	5.655137	-2.666507	0.857285
24	C	6.044219	-1.377228	0.447713
25	C	-5.823228	-2.129844	-0.585909
26	C	-5.347944	-3.468103	-0.387365
27	C	-3.992995	-3.695222	-0.002666
28	C	-3.091920	-2.668863	0.092070
29	C	-3.329106	2.201779	-0.716353
30	C	-4.341195	3.103635	-1.002291
31	C	-5.655146	2.666523	-0.857244
32	C	-6.044222	1.377236	-0.447692
33	O	-6.739221	3.494944	-1.012086
34	C	-7.822736	2.775379	-0.598839
35	C	-7.470955	1.493633	-0.174290
36	C	-9.123104	3.297745	-0.568747
37	C	-10.099150	2.511208	-0.012193
38	C	-9.792150	1.252642	0.584591
39	C	-8.452625	0.736465	0.546515
40	C	-7.114673	-1.957913	-1.139064
41	C	-7.921492	-3.039216	-1.417338
42	C	-7.483220	-4.350058	-1.146469
43	C	-6.217638	-4.554987	-0.647278
44	C	7.114672	1.957922	1.139072
45	C	7.921489	3.039226	1.417349
46	C	7.483226	4.350066	1.146459
47	C	6.217652	4.554995	0.647247
48	O	6.739208	-3.494927	1.012146
49	C	7.822726	-2.775375	0.598888
50	C	7.470950	-1.493638	0.174312

51	C	9.123094	-3.297743	0.568811
52	C	10.099142	-2.511219	0.012241
53	C	9.792145	-1.252666	-0.584572
54	C	8.452620	-0.736487	-0.546509
55	C	10.786130	-0.528634	-1.289453
56	C	10.474276	0.615828	-1.984615
57	C	9.142952	1.077356	-2.011433
58	C	8.159294	0.421027	-1.306205
59	C	-10.786135	0.528592	1.289454
60	C	-10.474281	-0.615888	1.984587
61	C	-9.142957	-1.077417	2.011392
62	C	-8.159300	-0.421069	1.306180
63	H	-1.038782	-1.387680	-1.768623
64	H	1.432743	-1.219427	-1.609620
65	H	1.038788	1.387694	1.768605
66	H	-1.432736	1.219439	1.609603
67	H	3.668204	4.716372	-0.165115
68	H	2.050780	2.856185	-0.321943
69	H	2.290704	-2.500532	0.773407
70	H	4.132574	-4.125000	1.292411
71	H	-3.668178	-4.716364	0.165053
72	H	-2.050763	-2.856172	0.321890
73	H	-2.290714	2.500558	-0.773398
74	H	-4.132593	4.125027	-1.292361
75	H	-9.324746	4.286871	-0.959714
76	H	-11.122731	2.866580	0.030755
77	H	-7.463138	-0.960999	-1.369531
78	H	-8.902712	-2.877381	-1.848836
79	H	-8.132546	-5.192855	-1.354338
80	H	-5.854409	-5.562487	-0.471743
81	H	7.463130	0.961009	1.369556
82	H	8.902702	2.877394	1.848864
83	H	8.132550	5.192864	1.354331
84	H	5.854429	5.562494	0.471698
85	H	9.324734	-4.286861	0.959797
86	H	11.122723	-2.866594	-0.030698
87	H	11.800732	-0.913453	-1.294569
88	H	11.243481	1.149941	-2.530770
89	H	8.889344	1.957240	-2.591565
90	H	7.141080	0.780686	-1.349499

91	H	-11.800737	0.913411	1.294579
92	H	-11.243487	-1.150015	2.530728
93	H	-8.889348	-1.957315	2.591502
94	H	-7.141086	-0.780730	1.349466

(*P*,*M*)**-3** optimized at wB97XD/6-311G(d,p) in gas phase*; E* = -2489.196384 Hartree

1	C	-1.384812	-0.086657	-0.075128
2	C	-0.592857	-0.801495	-0.969750
3	C	0.788528	-0.715700	-0.893731
4	C	1.384819	0.086684	0.075148
5	C	0.592863	0.801522	0.969772
6	C	-0.788522	0.715729	0.893750
7	N	2.795085	0.167390	0.152041
8	N	-2.795079	-0.167374	-0.152017
9	C	3.653838	-0.904062	0.362113
10	C	4.982754	-0.427793	0.353962
11	C	4.894946	1.014276	0.301638
12	C	3.547270	1.324579	0.110329
13	C	-3.653839	0.904053	-0.362180
14	C	-4.982752	0.427773	-0.354004
15	C	-4.894929	-1.014288	-0.301546
16	C	-3.547249	-1.324568	-0.110223
17	C	5.807525	2.081188	0.577498
18	C	5.352380	3.413278	0.377706
19	C	3.997726	3.656021	-0.001230
20	C	3.089252	2.645216	-0.094336
21	C	3.302499	-2.228916	0.651705
22	C	4.308245	-3.133680	0.919849
23	C	5.623825	-2.695966	0.787814
24	C	6.005117	-1.405307	0.410420
25	C	-5.807505	-2.081228	-0.577304
26	C	-5.352344	-3.413299	-0.377417
27	C	-3.997680	-3.656005	0.001509
28	C	-3.089214	-2.645185	0.094527
29	C	-3.302509	2.228889	-0.651863
30	C	-4.308260	3.133621	-0.920099
31	C	-5.623838	2.695903	-0.788061
32	C	-6.005125	1.405272	-0.410560
33	O	-6.704577	3.517109	-0.932809
34	C	-7.780924	2.785884	-0.550555

35	C	-7.431521	1.506340	-0.152811
36	C	-9.090279	3.290784	-0.528834
37	C	-10.059264	2.478220	-0.015290
38	C	-9.745604	1.208644	0.554861
39	C	-8.408183	0.721998	0.534947
40	C	-7.090735	-1.886246	-1.135382
41	C	-7.914601	-2.949878	-1.399453
42	C	-7.497687	-4.263895	-1.118244
43	C	-6.237317	-4.487149	-0.627719
44	C	7.090741	1.886160	1.135594
45	C	7.914609	2.949766	1.399766
46	C	7.497709	4.263807	1.118647
47	C	6.237353	4.487105	0.628111
48	O	6.704563	-3.517191	0.932457
49	C	7.780914	-2.785948	0.550251
50	C	7.431512	-1.506360	0.152650
51	C	9.090265	-3.290858	0.528450
52	C	10.059247	-2.478250	0.014971
53	C	9.745585	-1.208609	-0.555034
54	C	8.408168	-0.721953	-0.535039
55	C	10.740089	-0.447343	-1.214226
56	C	10.420222	0.706511	-1.879543
57	C	9.080922	1.138096	-1.931109
58	C	8.100006	0.441736	-1.275031
59	C	-10.740117	0.447447	1.214120
60	C	-10.420257	-0.706326	1.879579
61	C	-9.080955	-1.137893	1.931224
62	C	-8.100031	-0.441601	1.275085
63	H	-1.065584	-1.407129	-1.733658
64	H	1.413617	-1.254555	-1.595760
65	H	1.065588	1.407152	1.733684
66	H	-1.413612	1.254588	1.595775
67	H	3.684285	4.681309	-0.166387
68	H	2.048568	2.840833	-0.322005
69	H	2.262333	-2.525530	0.700788
70	H	4.096690	-4.160264	1.188440
71	H	-3.684228	-4.681279	0.166732
72	H	-2.048522	-2.840773	0.322191
73	H	-2.262346	2.525507	-0.700954
74	H	-4.096712	4.160185	-1.188772

75	H	-9.301007	4.285479	-0.899852
76	H	-11.089320	2.816427	0.015581
77	H	-7.417509	-0.882709	-1.373196
78	H	-8.894513	-2.774579	-1.828178
79	H	-8.162598	-5.096794	-1.315660
80	H	-5.890171	-5.499691	-0.448106
81	H	7.417502	0.882608	1.373346
82	H	8.894509	2.774430	1.828504
83	H	8.162620	5.096687	1.316143
84	H	5.890216	5.499663	0.448569
85	H	9.300989	-4.285594	0.899360
86	H	11.089300	-2.816462	-0.015958
87	H	11.762988	-0.809750	-1.205241
88	H	11.190629	1.273792	-2.389028
89	H	8.821958	2.027651	-2.493513
90	H	7.071885	0.772458	-1.335669
91	H	-11.763018	0.809846	1.205073
92	H	-11.190669	-1.273551	2.389119
93	H	-8.821995	-2.027379	2.493741
94	H	-7.071912	-0.772303	1.335794

(*P*,*M*)-**3** optimized at MN15/6-311G(d,p) in gas phase; *E* = -2487.037400 Hartree

1	C	-1.387951	-0.091964	-0.080515
2	C	-0.586493	-0.870165	-0.916855
3	C	0.796597	-0.779228	-0.835222
4	C	1.387948	0.091945	0.080427
5	C	0.586491	0.870146	0.916768
6	C	-0.796599	0.779208	0.835136
7	N	2.799009	0.177604	0.161805
8	N	-2.799010	-0.177620	-0.161897
9	C	3.663655	-0.894200	0.371258
10	C	4.995365	-0.415035	0.359568
11	C	4.903318	1.028041	0.308082
12	C	3.551418	1.338639	0.118468
13	C	-3.663657	0.894191	-0.371316
14	C	-4.995368	0.415033	-0.359593
15	C	-4.903328	-1.028044	-0.308123
16	C	-3.551426	-1.338651	-0.118545
17	C	5.815755	2.098050	0.579735
18	C	5.357211	3.431972	0.382216

19	C	4.002540	3.674494	0.001076
20	C	3.092655	2.659803	-0.092808
21	C	3.310453	-2.217381	0.673931
22	C	4.318129	-3.122891	0.947440
23	C	5.634827	-2.686666	0.807346
24	C	6.020906	-1.395990	0.419823
25	C	-5.815776	-2.098045	-0.579771
26	C	-5.357234	-3.431972	-0.382274
27	C	-4.002556	-3.674504	-0.001164
28	C	-3.092664	-2.659819	0.092708
29	C	-3.310458	2.217373	-0.673990
30	C	-4.318138	3.122888	-0.947468
31	C	-5.634835	2.686669	-0.807339
32	C	-6.020907	1.395993	-0.419811
33	O	-6.714614	3.510192	-0.954001
34	C	-7.795354	2.787775	-0.563063
35	C	-7.447146	1.505550	-0.158870
36	C	-9.103288	3.300331	-0.537412
37	C	-10.075742	2.494286	-0.011076
38	C	-9.763454	1.225516	0.563410
39	C	-8.427102	0.729244	0.536912
40	C	-7.103527	-1.903705	-1.131278
41	C	-7.928278	-2.971215	-1.396095
42	C	-7.506094	-4.287512	-1.120088
43	C	-6.240941	-4.508660	-0.632712
44	C	7.103496	1.903719	1.131267
45	C	7.928237	2.971235	1.396093
46	C	7.506052	4.287527	1.120067
47	C	6.240908	4.508666	0.632662
48	O	6.714606	-3.510186	0.954040
49	C	7.795354	-2.787767	0.563128
50	C	7.447153	-1.505544	0.158923
51	C	9.103289	-3.300321	0.537511
52	C	10.075755	-2.494277	0.011197
53	C	9.763480	-1.225509	-0.563300
54	C	8.427127	-0.729238	-0.536836
55	C	10.759768	-0.474176	-1.233974
56	C	10.443467	0.681025	-1.904752
57	C	9.104630	1.123136	-1.948485
58	C	8.121351	0.434868	-1.279672

59	C	-10.759726	0.474181	1.234107
60	C	-10.443409	-0.681021	1.904873
61	C	-9.104571	-1.123133	1.948573
62	C	-8.121307	-0.434864	1.279738
63	H	-1.056334	-1.523790	-1.642866
64	H	1.430284	-1.362405	-1.493770
65	H	1.056332	1.523768	1.642781
66	H	-1.430286	1.362383	1.493685
67	H	3.693021	4.701002	-0.166022
68	H	2.052569	2.851963	-0.327910
69	H	2.268800	-2.508451	0.732658
70	H	4.110423	-4.147608	1.226064
71	H	-3.693040	-4.701015	0.165919
72	H	-2.052575	-2.851986	0.327789
73	H	-2.268806	2.508440	-0.732743
74	H	-4.110435	4.147606	-1.226091
75	H	-9.307540	4.294725	-0.912891
76	H	-11.105282	2.834211	0.027763
77	H	-7.428567	-0.897074	-1.365318
78	H	-8.909915	-2.797828	-1.821758
79	H	-8.169422	-5.121423	-1.318222
80	H	-5.886697	-5.519460	-0.455183
81	H	7.428538	0.897091	1.365317
82	H	8.909865	2.797857	1.821779
83	H	8.169372	5.121443	1.318207
84	H	5.886666	5.519463	0.455116
85	H	9.307532	-4.294714	0.912998
86	H	11.105296	-2.834202	-0.027615
87	H	11.778979	-0.847862	-1.228458
88	H	11.214028	1.239715	-2.423283
89	H	8.848307	2.011033	-2.514761
90	H	7.091832	0.767579	-1.333260
91	H	-11.778937	0.847869	1.228618
92	H	-11.213957	-1.239712	2.423423
93	H	-8.848235	-2.011033	2.514839
94	H	-7.091787	-0.767574	1.333302

(*M*,*M*)**-3** optimized at MN15/6-311G(d,p) in gas phase; *E* = -2487.036572 Hartree

1	C	-4.299399	-3.222432	-0.409466
2	C	-5.613904	-2.784894	-0.253829

3	C	-6.002670	-1.454254	-0.045618
4	C	-4.996487	-0.464088	-0.203232
5	C	-3.658187	-0.923975	-0.226762
6	C	-3.296282	-2.273945	-0.344122
7	C	-4.932169	0.970445	-0.380815
8	C	-3.577962	1.322204	-0.329009
9	N	-2.805280	0.177170	-0.240993
10	C	-5.882197	1.974842	-0.754802
11	C	-5.440590	3.328319	-0.795420
12	C	-4.070303	3.642505	-0.544811
13	C	-3.134915	2.665156	-0.354613
14	O	-6.682931	-3.633463	-0.202416
15	C	-7.752679	-2.871622	0.141010
16	C	-7.407424	-1.538303	0.319983
17	C	-9.045377	-3.389037	0.328481
18	C	-10.000594	-2.522283	0.785287
19	C	-9.680553	-1.176401	1.136535
20	C	-8.359104	-0.674805	0.949128
21	C	-10.649908	-0.341816	1.745344
22	C	-10.318061	0.906980	2.208764
23	C	-8.988715	1.365975	2.098864
24	C	-8.033524	0.593823	1.483015
25	C	-7.197125	1.682756	-1.185810
26	C	-8.058959	2.686871	-1.558671
27	C	-7.648405	4.034754	-1.516683
28	C	-6.360510	4.343077	-1.151502
29	C	-1.390358	0.120668	-0.240742
30	C	-0.660745	0.763756	-1.241374
31	C	0.726577	0.700592	-1.239664
32	C	1.392571	-0.006469	-0.238388
33	C	0.662951	-0.652339	0.759749
34	C	-0.724162	-0.589739	0.758505
35	N	2.807783	-0.082205	-0.238498
36	C	3.686656	0.997787	-0.216700
37	C	5.013437	0.502546	-0.202170
38	C	4.912704	-0.928881	-0.392581
39	C	3.550251	-1.246609	-0.340319
40	C	3.358400	2.360487	-0.240339
41	C	4.386078	3.284875	-0.270372
42	C	5.690218	2.806470	-0.161499

43	C	6.044691	1.458534	-0.007147
44	C	5.832919	-1.943172	-0.810611
45	C	5.353763	-3.281187	-0.905549
46	C	3.971354	-3.563869	-0.689642
47	C	3.064092	-2.568918	-0.458032
48	O	6.781529	3.624403	-0.084003
49	C	7.831091	2.822757	0.229603
50	C	7.451084	1.493581	0.363077
51	C	9.137311	3.299261	0.431363
52	C	10.069970	2.392945	0.856789
53	C	9.714524	1.045347	1.164644
54	C	8.379970	0.585630	0.963353
55	C	7.154049	-1.671014	-1.236626
56	C	7.987812	-2.683358	-1.648328
57	C	7.541563	-4.020723	-1.654269
58	C	6.245883	-4.307001	-1.299777
59	C	10.661603	0.166405	1.745499
60	C	10.296599	-1.086723	2.170715
61	C	8.955127	-1.505670	2.049451
62	C	8.020732	-0.689363	1.459226
63	H	-4.087470	-4.275484	-0.539807
64	H	-2.254326	-2.558636	-0.424662
65	H	-3.772896	4.685966	-0.558666
66	H	-2.086420	2.903089	-0.220099
67	H	-9.251110	-4.431867	0.124269
68	H	-11.018456	-2.863713	0.941490
69	H	-11.659356	-0.723724	1.862098
70	H	-11.067303	1.530333	2.682924
71	H	-8.717294	2.333826	2.504230
72	H	-7.009960	0.942782	1.419388
73	H	-7.514735	0.648426	-1.240500
74	H	-9.061098	2.438399	-1.888575
75	H	-8.340084	4.820289	-1.798061
76	H	-6.017314	5.373087	-1.155123
77	H	-1.190125	1.287745	-2.028981
78	H	1.303433	1.173981	-2.025988
79	H	1.194087	-1.182228	1.542452
80	H	-1.302368	-1.069975	1.539755
81	H	2.323759	2.679708	-0.252739
82	H	4.200322	4.349371	-0.327393

83	H	3.639927	-4.593466	-0.776926
84	H	2.003613	-2.777321	-0.382681
85	H	9.370032	4.342658	0.262015
86	H	11.096672	2.702056	1.022156
87	H	7.499486	-0.644429	-1.254738
88	H	8.995420	-2.449723	-1.972392
89	H	8.211484	-4.813598	-1.966037
90	H	5.873637	-5.325912	-1.344111
91	H	11.681024	0.517308	1.872452
92	H	11.029169	-1.744421	2.624079
93	H	8.657777	-2.477632	2.425766
94	H	6.987889	-1.007900	1.387669

(*P*,*P*)-**3** optimized at MN15/6-311G(d,p) in gas phase; *E* = -2487.036572 Hartree

1	C	4.299416	-3.222443	-0.409503
2	C	5.613915	-2.784900	-0.253836
3	C	6.002672	-1.454257	-0.045617
4	C	4.996488	-0.464096	-0.203253
5	C	3.658191	-0.923988	-0.226810
6	C	3.296294	-2.273959	-0.344181
7	C	4.932168	0.970438	-0.380829
8	C	3.577959	1.322191	-0.329047
9	N	2.805280	0.177153	-0.241055
10	C	5.882198	1.974838	-0.754801
11	C	5.440588	3.328314	-0.795421
12	C	4.070296	3.642495	-0.544830
13	C	3.134908	2.665142	-0.354651
14	O	6.682945	-3.633464	-0.202402
15	C	7.752684	-2.871618	0.141041
16	C	7.407420	-1.538301	0.320006
17	C	9.045381	-3.389028	0.328532
18	C	10.000588	-2.522269	0.785350
19	C	9.680537	-1.176387	1.136589
20	C	8.359088	-0.674798	0.949163
21	C	10.649880	-0.341797	1.745410
22	C	10.318020	0.906997	2.208826
23	C	8.988673	1.365986	2.098908
24	C	8.033494	0.593829	1.483044
25	C	7.197133	1.682757	-1.185792
26	C	8.058969	2.686875	-1.558640

27	C	7.648409	4.034757	-1.516654
28	C	6.360510	4.343075	-1.151489
29	C	1.390357	0.120648	-0.240809
30	C	0.660748	0.763746	-1.241438
31	C	-0.726574	0.700583	-1.239731
32	C	-1.392570	-0.006487	-0.238463
33	C	-0.662954	-0.652368	0.759670
34	C	0.724159	-0.589770	0.758429
35	N	-2.807783	-0.082217	-0.238575
36	C	-3.686652	0.997779	-0.216754
37	C	-5.013434	0.502543	-0.202197
38	C	-4.912710	-0.928884	-0.392611
39	C	-3.550258	-1.246618	-0.340379
40	C	-3.358389	2.360477	-0.240400
41	C	-4.386064	3.284870	-0.270414
42	C	-5.690204	2.806470	-0.161518
43	C	-6.044681	1.458537	-0.007159
44	C	-5.832940	-1.943173	-0.810612
45	C	-5.353794	-3.281191	-0.905550
46	C	-3.971380	-3.563878	-0.689678
47	C	-3.064108	-2.568930	-0.458098
48	O	-6.781510	3.624409	-0.084002
49	C	-7.831070	2.822767	0.229624
50	C	-7.451066	1.493590	0.363091
51	C	-9.137284	3.299277	0.431407
52	C	-10.069938	2.392966	0.856852
53	C	-9.714492	1.045367	1.164704
54	C	-8.379944	0.585644	0.963388
55	C	-7.154078	-1.671011	-1.236601
56	C	-7.987856	-2.683353	-1.648277
57	C	-7.541615	-4.020721	-1.654213
58	C	-6.245929	-4.307004	-1.299747
59	C	-10.661563	0.166431	1.745580
60	C	-10.296554	-1.086695	2.170798
61	C	-8.955086	-1.505647	2.049510
62	C	-8.020700	-0.689347	1.459260
63	H	4.087495	-4.275495	-0.539851
64	H	2.254340	-2.558654	-0.424744
65	H	3.772887	4.685955	-0.558682
66	H	2.086411	2.903071	-0.220152

67	H	9.251120	-4.431858	0.124327
68	H	11.018450	-2.863694	0.941567
69	H	11.659328	-0.723699	1.862177
70	H	11.067252	1.530355	2.682995
71	H	8.717241	2.333835	2.504270
72	H	7.009929	0.942783	1.419402
73	H	7.514747	0.648428	-1.240478
74	H	9.061112	2.438407	-1.888533
75	H	8.340090	4.820295	-1.798021
76	H	6.017311	5.373085	-1.155111
77	H	1.190132	1.287741	-2.029037
78	H	-1.303430	1.173980	-2.026051
79	H	-1.194094	-1.182267	1.542363
80	H	1.302364	-1.070015	1.539674
81	H	-2.323747	2.679693	-0.252820
82	H	-4.200304	4.349365	-0.327438
83	H	-3.639960	-4.593477	-0.776965
84	H	-2.003627	-2.777336	-0.382775
85	H	-9.370003	4.342675	0.262062
86	H	-11.096637	2.702080	1.022237
87	H	-7.499509	-0.644424	-1.254712
88	H	-8.995468	-2.449715	-1.972323
89	H	-8.211548	-4.813595	-1.965957
90	H	-5.873691	-5.325917	-1.344077
91	H	-11.680981	0.517337	1.872551
92	H	-11.029117	-1.744389	2.624178
93	H	-8.657732	-2.477607	2.425827
94	H	-6.987859	-1.007888	1.387683

TS-I calculated at MN15/6-311G(d,p) in gas phase*; E* = -2486.9966 Hartree

1	C	-1.420443	0.008733	-0.381031
2	C	-0.679961	-0.417508	-1.495657
3	C	0.713704	-0.337747	-1.483520
4	C	1.382946	0.171626	-0.360521
5	C	0.643457	0.598250	0.753319
6	C	-0.750170	0.517789	0.743084
7	N	2.808623	0.248025	-0.355244
8	N	-2.843044	-0.068960	-0.387808
9	C	3.675704	-0.848038	-0.316088
10	C	5.044149	-0.393456	-0.235047

11	C	4.944226	1.091074	-0.327515
12	C	3.564267	1.409247	-0.269313
13	C	-3.721220	1.007962	-0.171498
14	C	-5.064987	0.515735	-0.205582
15	C	-4.979988	-0.872787	-0.634788
16	C	-3.606299	-1.208841	-0.663903
17	C	5.827935	2.222614	-0.519762
18	C	5.300167	3.562134	-0.396591
19	C	3.903863	3.774755	-0.194466
20	C	3.033019	2.717579	-0.189196
21	C	3.213706	-2.159224	-0.115673
22	C	4.106769	-3.087869	0.376636
23	C	5.411802	-2.653227	0.580357
24	C	6.002955	-1.418869	0.183614
25	C	-5.922272	-1.819383	-1.168790
26	C	-5.448092	-3.147087	-1.467003
27	C	-4.065471	-3.471134	-1.291478
28	C	-3.141201	-2.516583	-0.934787
29	C	-3.384287	2.368187	-0.066914
30	C	-4.412213	3.300527	0.039764
31	C	-5.714334	2.812464	0.138523
32	C	-6.088759	1.448029	0.136077
33	O	-6.828130	3.636877	0.355469
34	C	-7.896470	2.773756	0.602809
35	C	-7.493666	1.432376	0.553697
36	C	-9.190102	3.217927	0.913324
37	C	-10.112854	2.255577	1.263651
38	C	-9.744833	0.878233	1.386236
39	C	-8.405508	0.445781	1.065107
40	C	-7.259217	-1.492757	-1.520370
41	C	-8.106849	-2.438394	-2.070903
42	C	-7.662552	-3.762596	-2.291006
43	C	-6.355071	-4.103165	-1.998861
44	C	7.168832	2.111273	-0.943039
45	C	7.987862	3.210380	-1.130299
46	C	7.491059	4.514447	-0.919171
47	C	6.164833	4.676625	-0.566938
48	O	6.277605	-3.436097	1.346191
49	C	7.463124	-2.726321	1.419245
50	C	7.428343	-1.578983	0.611986

51	C	8.512984	-3.120652	2.265265
52	C	9.655199	-2.357064	2.242910
53	C	9.816534	-1.334762	1.255981
54	C	8.734880	-1.009903	0.352276
55	C	11.092702	-0.740509	1.055068
56	C	11.361550	0.024939	-0.063351
57	C	10.362925	0.170443	-1.051571
58	C	9.092365	-0.340046	-0.841787
59	C	-10.671394	-0.073524	1.895178
60	C	-10.292299	-1.378561	2.143067
61	C	-8.957711	-1.780859	1.900899
62	C	-8.039277	-0.891676	1.372160
63	H	-1.198562	-0.786125	-2.373008
64	H	1.288390	-0.647624	-2.349136
65	H	1.163692	0.969926	1.628873
66	H	-1.322345	0.825561	1.610749
67	H	3.536686	4.792838	-0.109397
68	H	1.962422	2.867367	-0.121996
69	H	2.163695	-2.395504	-0.230116
70	H	3.813916	-4.087625	0.669977
71	H	-3.743160	-4.487194	-1.498310
72	H	-2.086643	-2.755193	-0.865244
73	H	-2.350393	2.687810	-0.106496
74	H	-4.219477	4.364855	0.081714
75	H	-9.429052	4.273727	0.886632
76	H	-11.131069	2.541569	1.508077
77	H	-7.610776	-0.479828	-1.375692
78	H	-9.121267	-2.159221	-2.336811
79	H	-8.340754	-4.499493	-2.708946
80	H	-5.990788	-5.106990	-2.199819
81	H	7.561665	1.130443	-1.102405
82	H	9.017560	3.062262	-1.440082
83	H	8.136010	5.376502	-1.054595
84	H	5.747563	5.672252	-0.442578
85	H	8.389301	-3.978868	2.913923
86	H	10.483705	-2.578532	2.907707
87	H	11.877481	-0.953616	1.775457
88	H	12.346950	0.452862	-0.214614
89	H	10.599800	0.653066	-1.994554
90	H	8.374351	-0.317365	-1.652332

91	H	-11.683436	0.255596	2.113985
92	H	-11.007480	-2.089661	2.543762
93	H	-8.650588	-2.795540	2.132486
94	H	-7.018439	-1.209594	1.207984

TS-II calculated at MN15/6-311G(d,p) in gas phase; E = -2486.99705 Hartree

1	C	-1.382376	-0.17445	0.353330
2	C	-0.641160	-0.603140	-0.758581
3	C	0.752398	-0.521825	-0.746718
4	C	1.420928	-0.009844	0.377109
5	C	0.678738	0.418402	1.489825
6	C	-0.714859	0.337779	1.476073
7	N	2.843467	0.068845	0.385500
8	N	-2.807948	-0.251598	0.346267
9	C	3.722676	-1.007902	0.172561
10	C	5.066042	-0.514617	0.207128
11	C	4.979535	0.874746	0.633287
12	C	3.605571	1.209862	0.660077
13	C	-3.675536	0.843954	0.307017
14	C	-5.043720	0.388744	0.225269
15	C	-4.943225	-1.096182	0.315917
16	C	-3.562844	-1.413242	0.259845
17	C	5.920508	1.823148	1.166395
18	C	5.445022	3.151139	1.461232
19	C	4.062376	3.473812	1.283390
20	C	3.139213	2.517839	0.927638
21	C	3.386863	-2.368595	0.070476
22	C	4.415596	-3.300400	-0.033058
23	C	5.717470	-2.811587	-0.131378
24	C	6.090887	-1.446874	-0.131399
25	C	-5.826807	-2.228537	0.505087
26	C	-5.295863	-3.567900	0.393441
27	C	-3.898595	-3.779628	0.197830
28	C	-3.029580	-2.721138	0.187010
29	C	-3.213751	2.155035	0.104720
30	C	-4.106885	3.083009	-0.388589
31	C	-5.412349	2.648522	-0.589763
32	C	-6.003101	1.414852	-0.190391
33	O	-6.280546	3.432245	-1.351976
34	C	-7.467911	2.724882	-1.417901

35	C	-7.430548	1.577535	-0.610730
36	C	-8.523005	3.122521	-2.255742
37	C	-9.667914	2.363317	-2.223577
38	C	-9.824828	1.341843	-1.235108
39	C	-8.736727	1.012660	-0.340790
40	C	-7.173177	-2.117750	0.910605
41	C	-7.991471	-3.217302	1.098059
42	C	-7.488968	-4.521850	0.904273
43	C	-6.159340	-4.683175	0.564914
44	C	7.257269	1.498226	1.520243
45	C	8.103573	2.445637	2.069766
46	C	7.658068	3.769984	2.286531
47	C	6.350689	4.108995	1.992127
48	O	6.832113	-3.635633	-0.345358
49	C	7.900093	-2.772246	-0.593321
50	C	7.496254	-1.431061	-0.547464
51	C	9.194394	-3.216115	-0.901468
52	C	10.116831	-2.253824	-1.252784
53	C	9.747944	-0.877007	-1.378650
54	C	8.407949	-0.444866	-1.059906
55	C	10.674375	0.074363	-1.888547
56	C	10.294610	1.378609	-2.139561
57	C	8.959465	1.780438	-1.899701
58	C	8.041090	0.891685	-1.370136
59	C	-11.101439	0.752233	-1.023547
60	C	-11.363386	-0.013380	0.096376
61	C	-10.356526	-0.164377	1.075404
62	C	-9.085944	0.341851	0.855270
63	H	-1.160027	-0.977050	-1.633996
64	H	1.325860	-0.831169	-1.612973
65	H	1.195969	0.789275	2.367034
66	H	-1.290809	0.649211	2.340291
67	H	3.739089	4.490077	1.487680
68	H	2.084567	2.755540	0.856345
69	H	2.353158	-2.688892	0.109538
70	H	4.223690	-4.364955	-0.072989
71	H	-3.529558	-4.797658	0.120521
72	H	-1.958622	-2.869609	0.122795
73	H	-2.163711	2.391471	0.218509
74	H	-3.814208	4.082439	-0.683208

75	H	-8.401621	3.980267	-2.905458
76	H	-10.501121	2.587802	-2.881444
77	H	-7.573048	-1.136947	1.051206
78	H	-9.025383	-3.068687	1.393355
79	H	-8.132918	-5.384510	1.040635
80	H	-5.738745	-5.678606	0.450568
81	H	7.609715	0.485241	1.378130
82	H	9.117876	2.167759	2.337464
83	H	8.335249	4.508252	2.703707
84	H	5.985452	5.112982	2.190526
85	H	9.434087	-4.271681	-0.872303
86	H	11.135525	-2.539579	-1.495482
87	H	11.686897	-0.254473	-2.105552
88	H	11.009720	2.089395	-2.540938
89	H	8.651863	2.794414	-2.133728
90	H	7.019841	1.209204	-1.207755
91	H	-11.891592	0.968775	-1.737000
92	H	-12.349051	-0.437713	0.255809
93	H	-10.586567	-0.648172	2.019471
94	H	-8.360291	0.313123	1.659041

8. References

1. Frisch, M.J.; Trucks, G.W.; Schlegel, H.B.; Scuseria, G.E.; Robb, M.A.; Cheeseman, J.R.; Scalmani, G.; Barone, V.; Petersson, G.A.; Nakatsuji, H.; et al. *Gaussian 16 Rev. C.01*, Wallingford, CT, **2016**.
2. Haoyu, S.Y.; He, X.; Li, S.L.; Truhlar, D.G. MN15: A Kohn–Sham global-hybrid exchange–correlation density functional with broad accuracy for multi-reference and single-reference systems and noncovalent interactions. *Chem. Sci.* **2016**, *7*, 5032-5051.
3. Casida, M.E.; Jamorski, C.; Casida, K.C.; Salahub, D.R. Molecular excitation energies to high-lying bound states from time-dependent density-functional response theory: Characterization and correction of the time-dependent local density approximation ionization threshold. *J. Chem. Phys.* **1998**, *108*, 4439– 4449.
4. Stratmann, R.E.; Scuseria, G.E.; Frisch, M.J. An efficient implementation of time-dependent density-functional theory for the calculation of excitation energies of large molecules. *J. Chem. Phys.* **1998**, *109*, 8218–8224.
5. Wolinski, K.; Hinton, J.F.; Pulay, P. Efficient implementation of the gauge-independent atomic orbital method for NMR chemical shift calculations. *J. Am. Chem. Soc.* **1990**, *112*, 8251–8260.
6. Bühl, M.; van Wüllen, C. Computational evidence for a new C84 isomer. *Chem. Phys. Lett.* **1995**, *247*, 63-68.
7. Schleyer, P.V.R.; Maerker, C.; Dransfeld, A.; Jiao, H.; van Eikema Hommes, N.J. Nucleus-independent chemical shifts: a simple and efficient aromaticity probe. *J. Am. Chem. Soc.* **1996**, *118*, 6317-6318.
